# Supplementary material for: Myofibroblast transcriptome indicates SFRP2hi fibroblast progenitors in systemic sclerosis skin
Source: Nat Commun. 2021 Jul 19;12:4384. doi: 10.1038/s41467-021-24607-6 (PMC8289865; doi:10.1038/s41467-021-24607-6)
Supplement: Supplementary file 1 — Supplementary Information [file 41467_2021_24607_MOESM1_ESM.pdf]

Supplementary Information

Supplementary Figure 1. Fibroblast populations in normal skin.
Supplementary Figure 2. Feature plots showing marker genes for each normal fibroblast subcluster.
Supplementary Figure 3 A and B. Heatmap of marker genes for normal fibroblast clusters.
Supplementary Figure 4. Immunofluorescent staining of normal and SSc skin identifying fibroblast subpopulations.
Supplementary Figure 5. Graphic of fibroblast heterogeneity in normal skin.
Supplementary Figure 6. Heatmap of genes most highly distinguishing each cluster in combined normal and SSc skin analysis.
Supplementary Figure 7. V1 and V2 chemistries in combined normal and SSc skin analysis show distribution across clusters.
Supplementary Figure 8. Feature plots showing marker genes for each cell type in combined normal and SSc skin analysis.
Supplementary Figure 9. UMAP plot of scRNA-seq data from control and SSc skin biopsies.
Supplementary Figure 10. UMAP plot of scRNA-seq data from control and SSc skin biopsies
Supplementary Figure 11. UMAP feature plots showing marker genes for each cell type in combined normal and SSc skin analysis
Supplementary Figure 12. Feature plots of key marker genes and proportions of fibroblast subclusters from control healthy and SSc skin.
Supplementary Figure 13. Fibroblast cell clusters lack markers, suggesting preadipocyte, pericyte or myeloid progenitors.
Supplementary Figure 14A . Immunofluorescent staining of control skin.
Supplementary Figure 14B. Immunofluorescent staining of SSc skin.
Supplementary Figure 15. Pseudotime modeling of SFRP2+ fibroblast differentiation in SSc skin.
Supplementary Figure 16. UMAP projection of Velocyto analysis.
Supplementary Figure 17. Analysis of myofibroblast regulons.
Supplementary Figure 18. Clustering of SFRP2+ regulons
Supplementary Figure 19. Analysis of regulons using filtered gene expression data.
Supplementary Figure 20. Analysis of regulons using restricted gene expression data.
Supplementary Figure 21. Foot-printed based regulon analysis by DoRothEA with high to low confidence levels of TF-target interactions.
Supplementary Figure 22. CHAC1 regulation through Smad3 phosphorylation.
Supplementary Figure 23. Feature plot showing module expression derived from DoRothEA.
Supplementary Figure 24. Feature plots showing module expression derived from SMAD3 siRNA treated myofibroblasts.

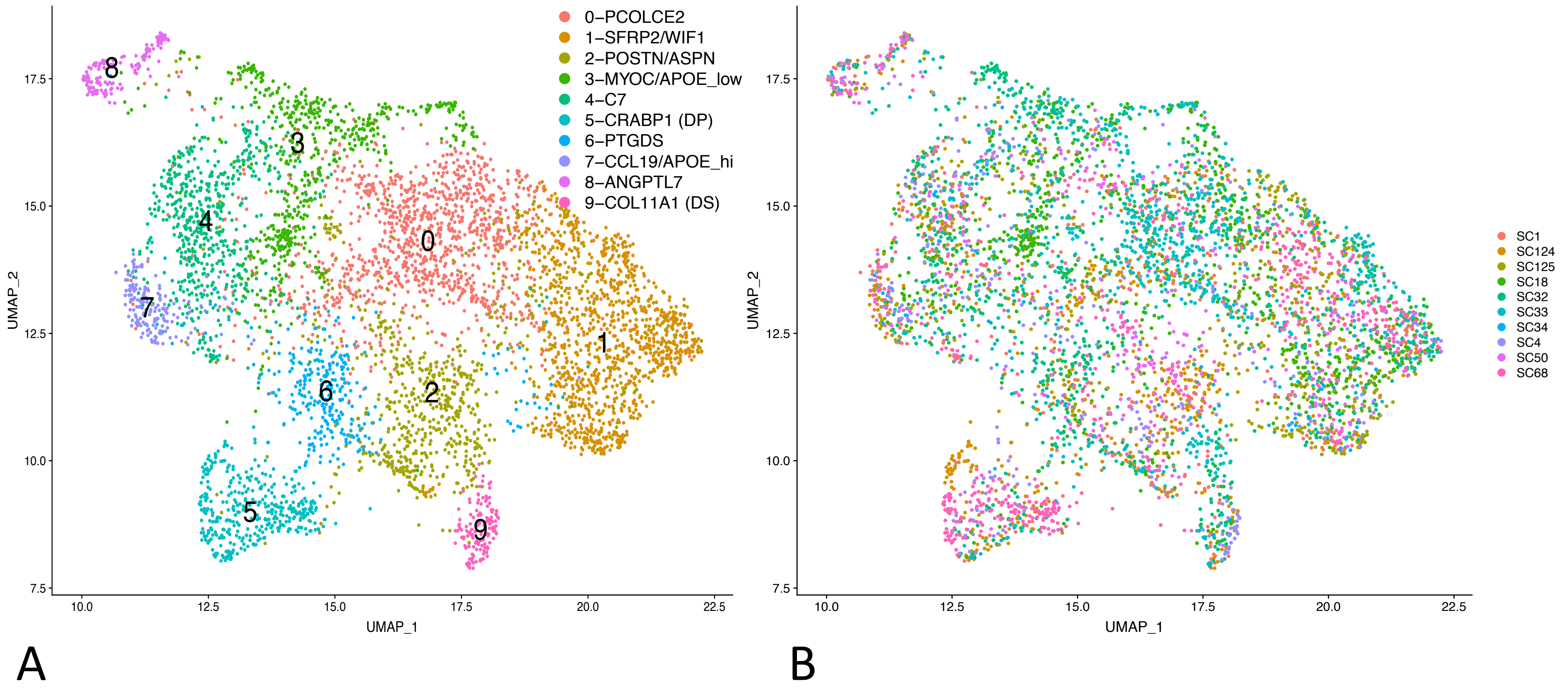

Supplementary Figure 1. Fibroblast populations in normal skin. UMAP clustering of cells colored by cluster (panel A) or by sample of origin (panel B).

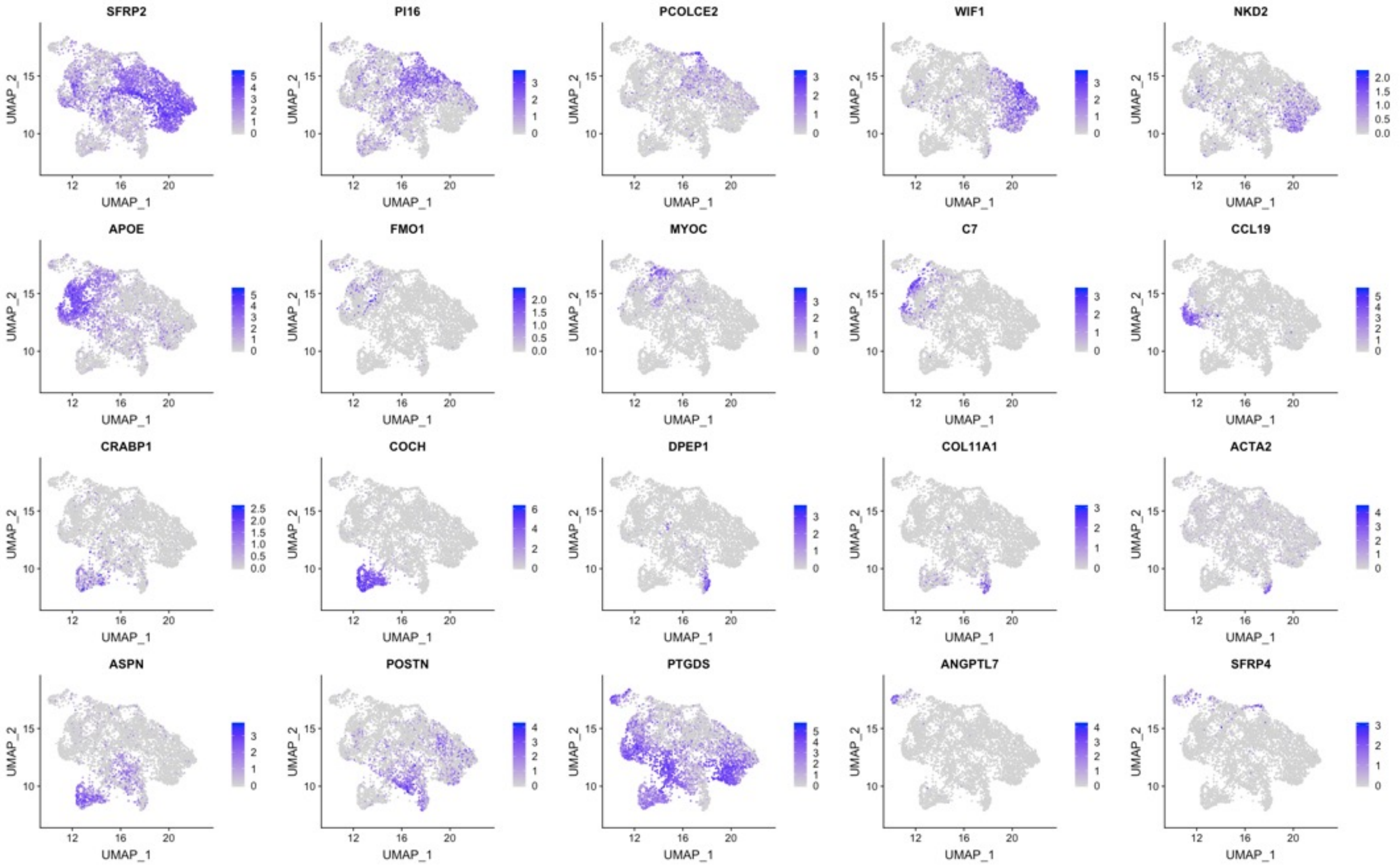

Supplementary Figure 2. Feature plots showing marker genes for each normal fibroblast subcluster. Purple coloration indicates level of expression

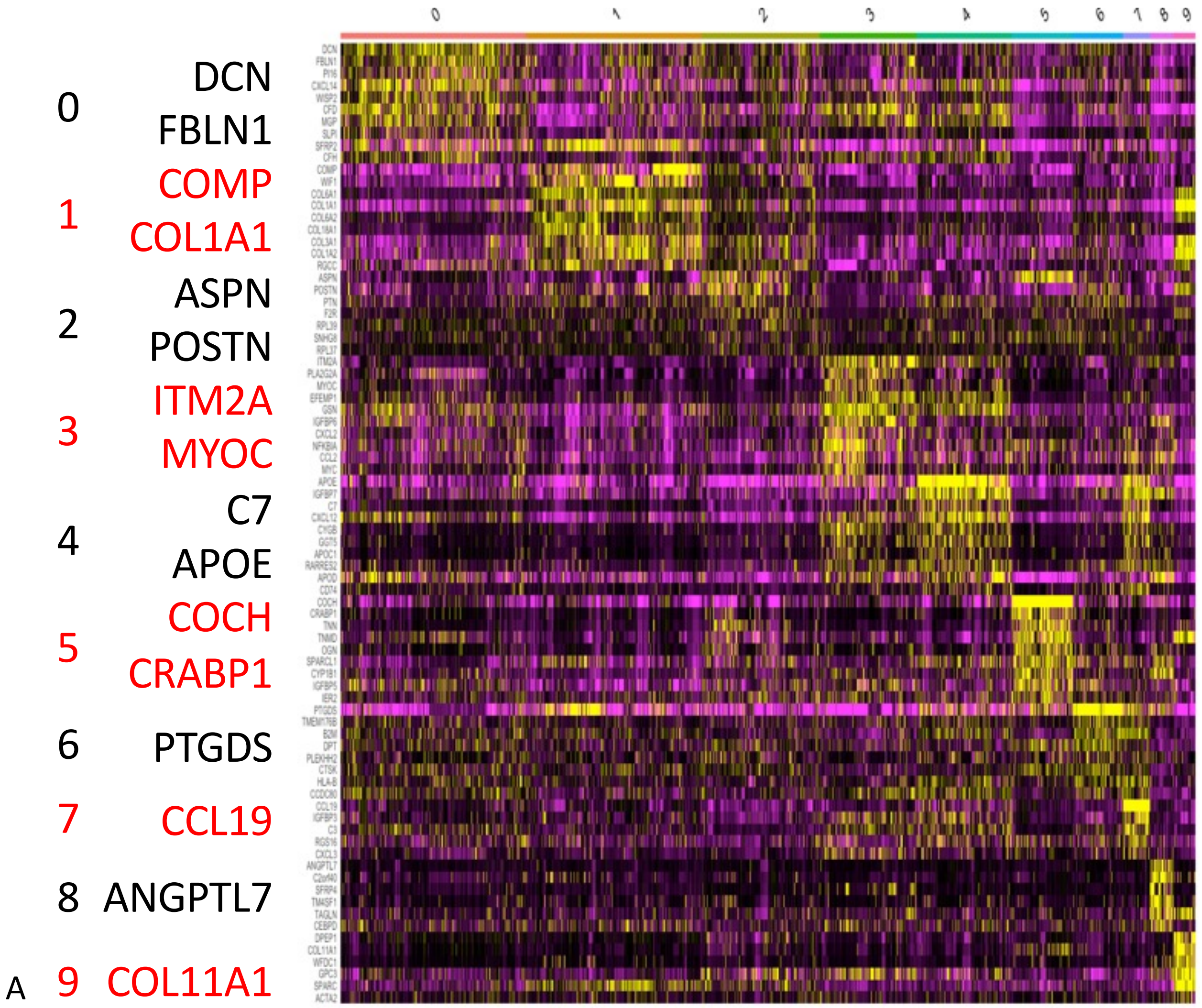

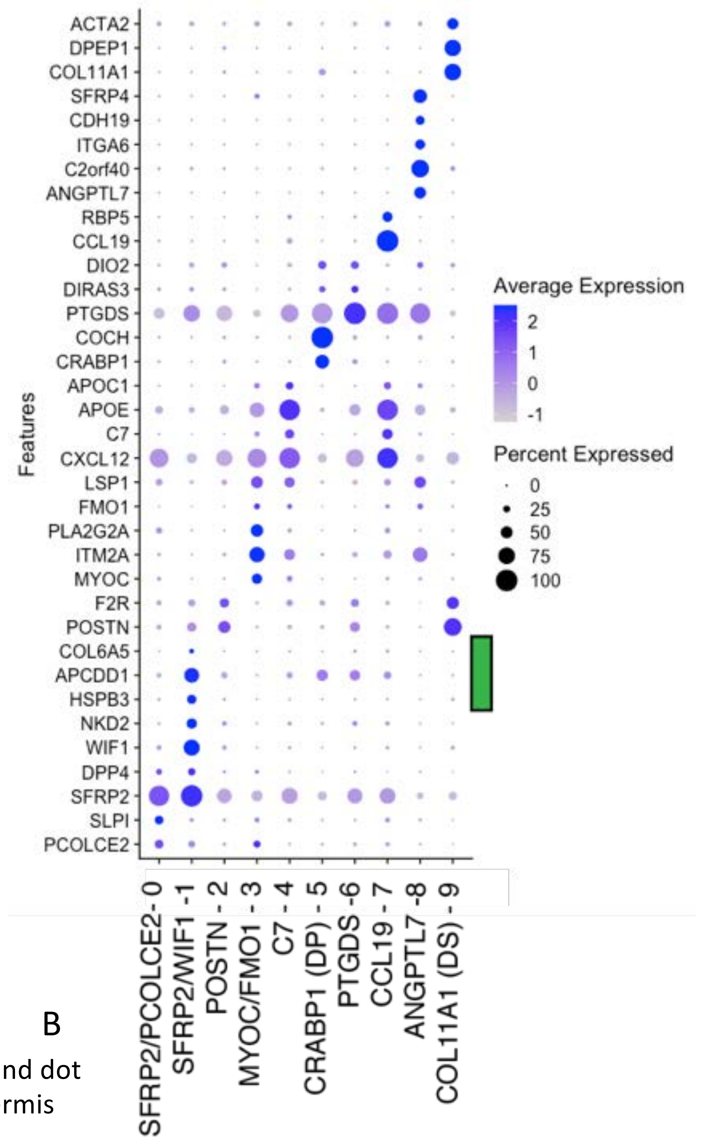

Supplementary Figure 3 A and B. Heatmap of marker genes for normal fibroblast clusters. Cluster (panel A) and dot plots (panel B) showing genes most differentially expressed in each cluster. Genes associated with papillary dermis are indicated by the green box.

Supplementary Figure 4. Immunofluorescent staining of normal and SSc skin identifying fibroblast subpopulations. CCL19 staining marks cells throughout the dermis (panels row a). CRABP1 staining marks dermal papilla fibroblasts at the base of a hair follicle (panels row b shown only in SSc skin), POSTN expressing prominently within the extracellular matrix in the papillary dermis (panels row c); SLPI staining cells in the deep dermis (panels row d). Slides were imaged at low and high magnification, scale bars mark 500µm and 100µm respectively. For all panels, nuclei are counterstained with DAPI (blue). Staining representative of n=3

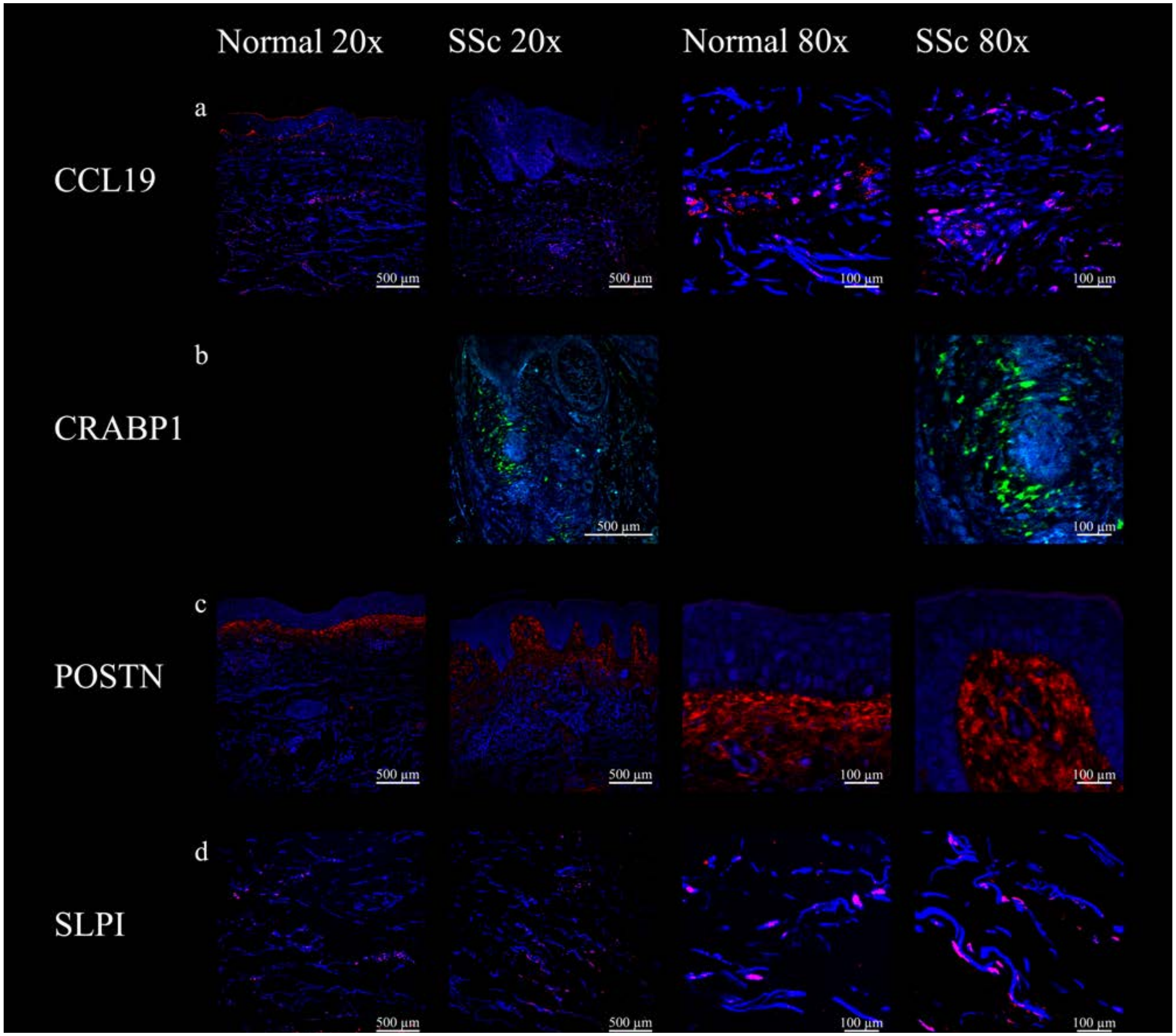

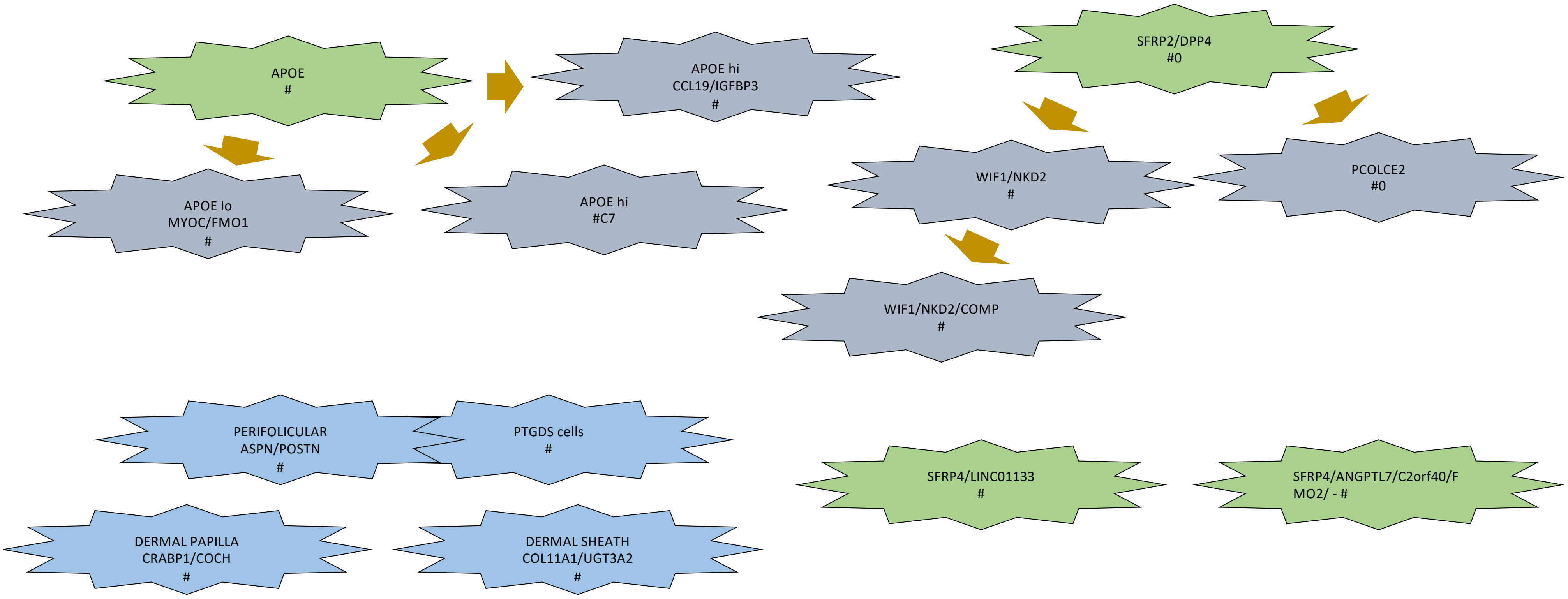

Supplementary Figure 5. Graphic of fibroblast heterogeneity in normal skin. Green and blue cells indicate primary clusters, and purple indicates subclusters within primary clusters, as indicated by arrows.

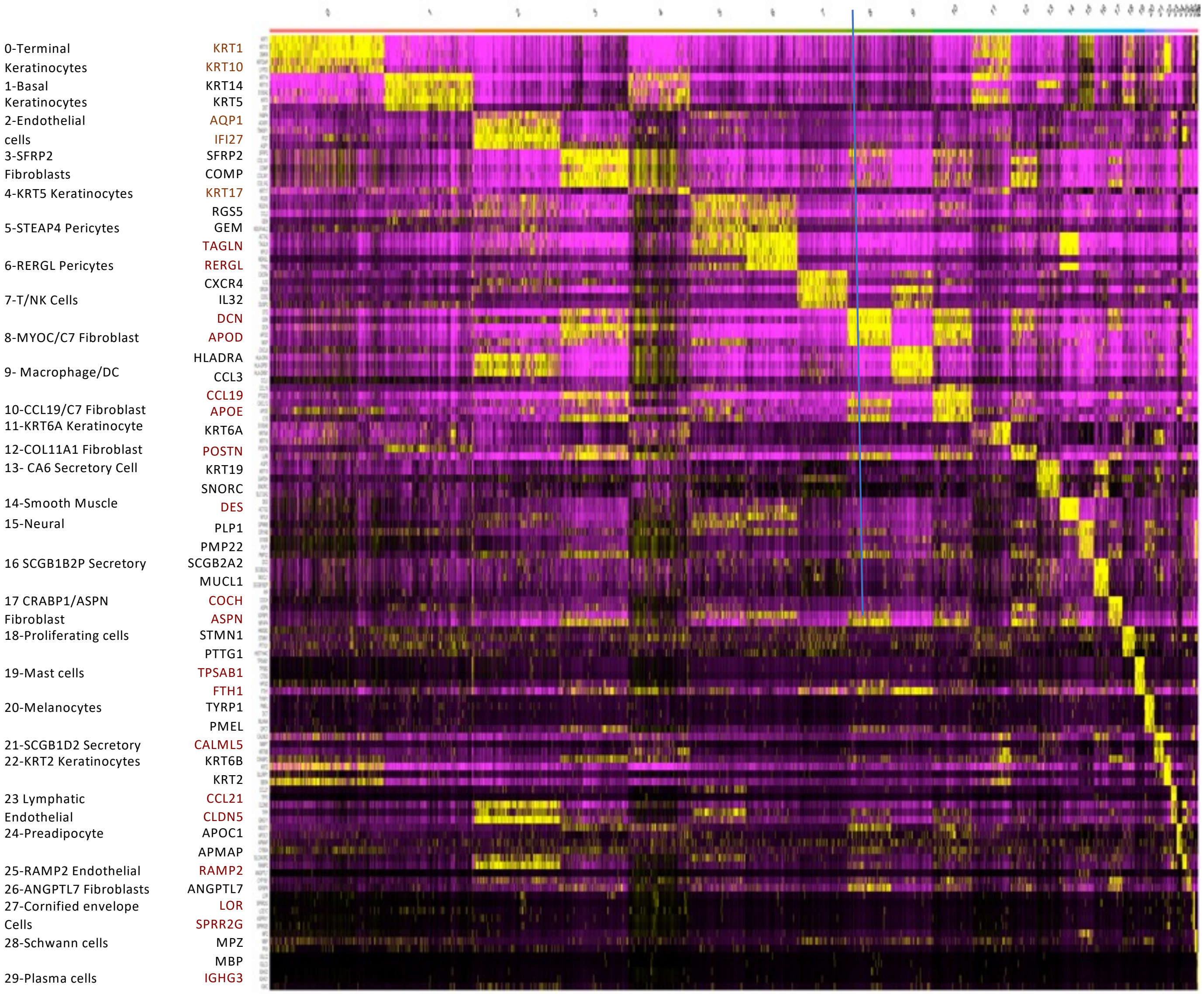

Supplementary Figure 6. Heatmap of genes most highly distinguishing each cluster in combined normal and SSc skin analysis. Informative marker genes are shown expanded to the left

Supplementary Figure 7. V1 and V2 chemistries in combined normal and SSc skin analysis show distribution across clusters.

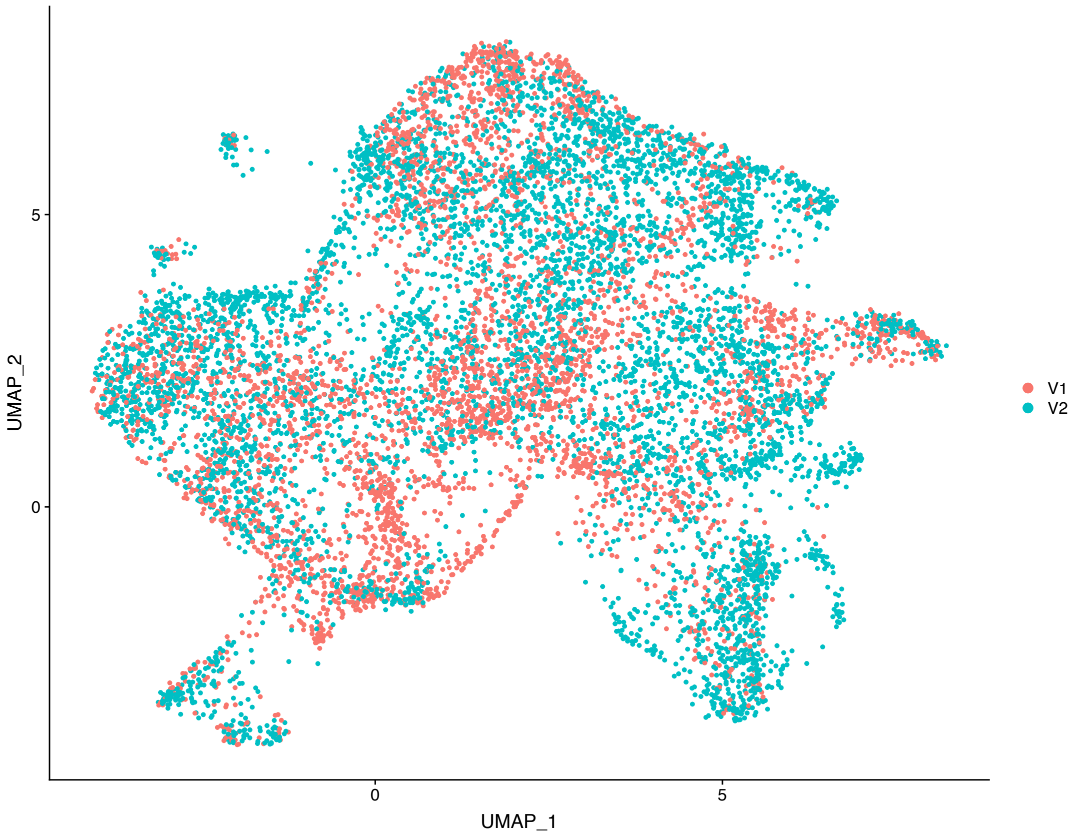

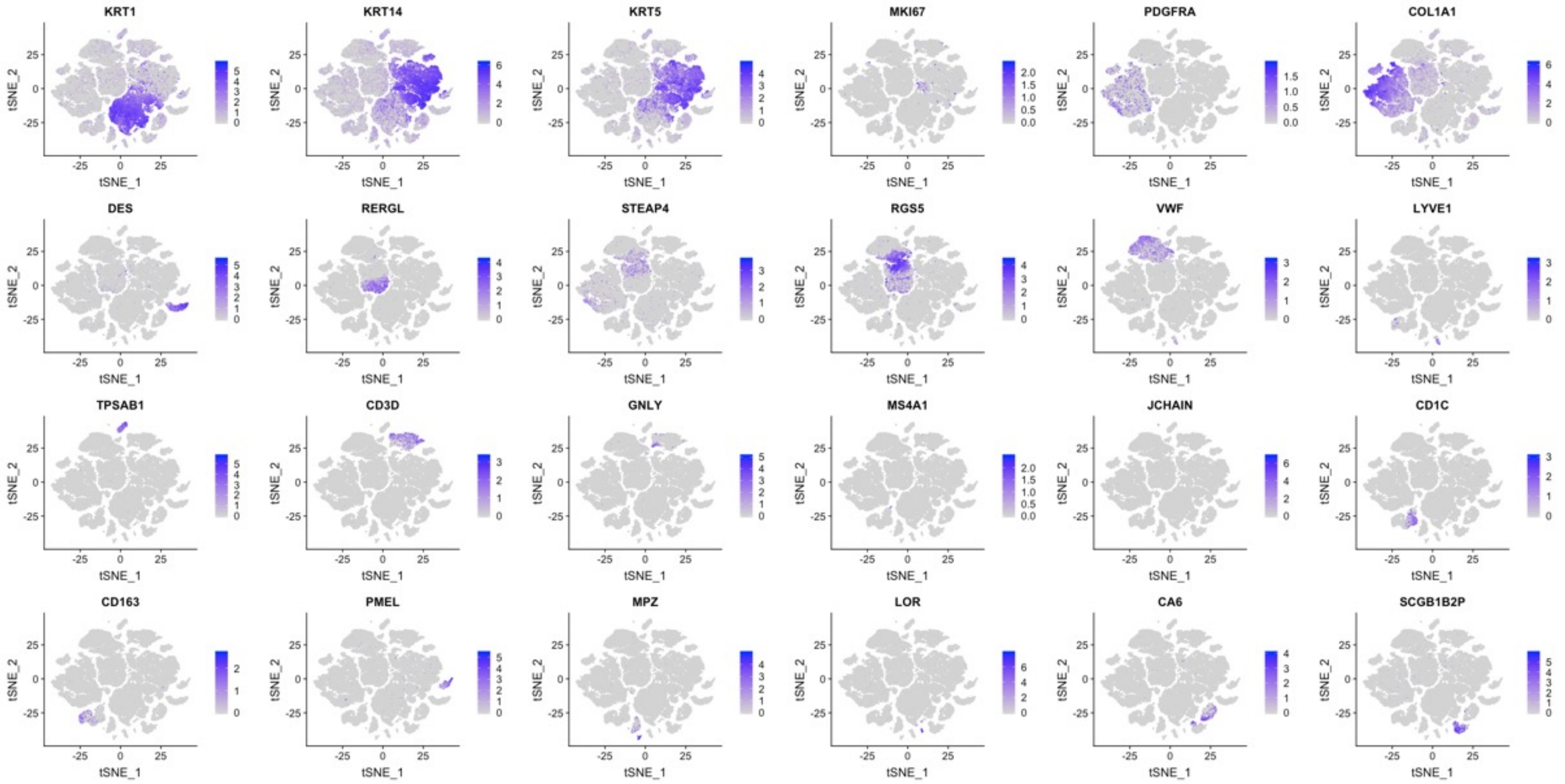

Supplementary Figure 8. Feature plots showing marker genes for each cell type in combined normal and SSc skin analysis. T-SNE plots are as in Figure 1. Purple coloration indicates the level of expression.

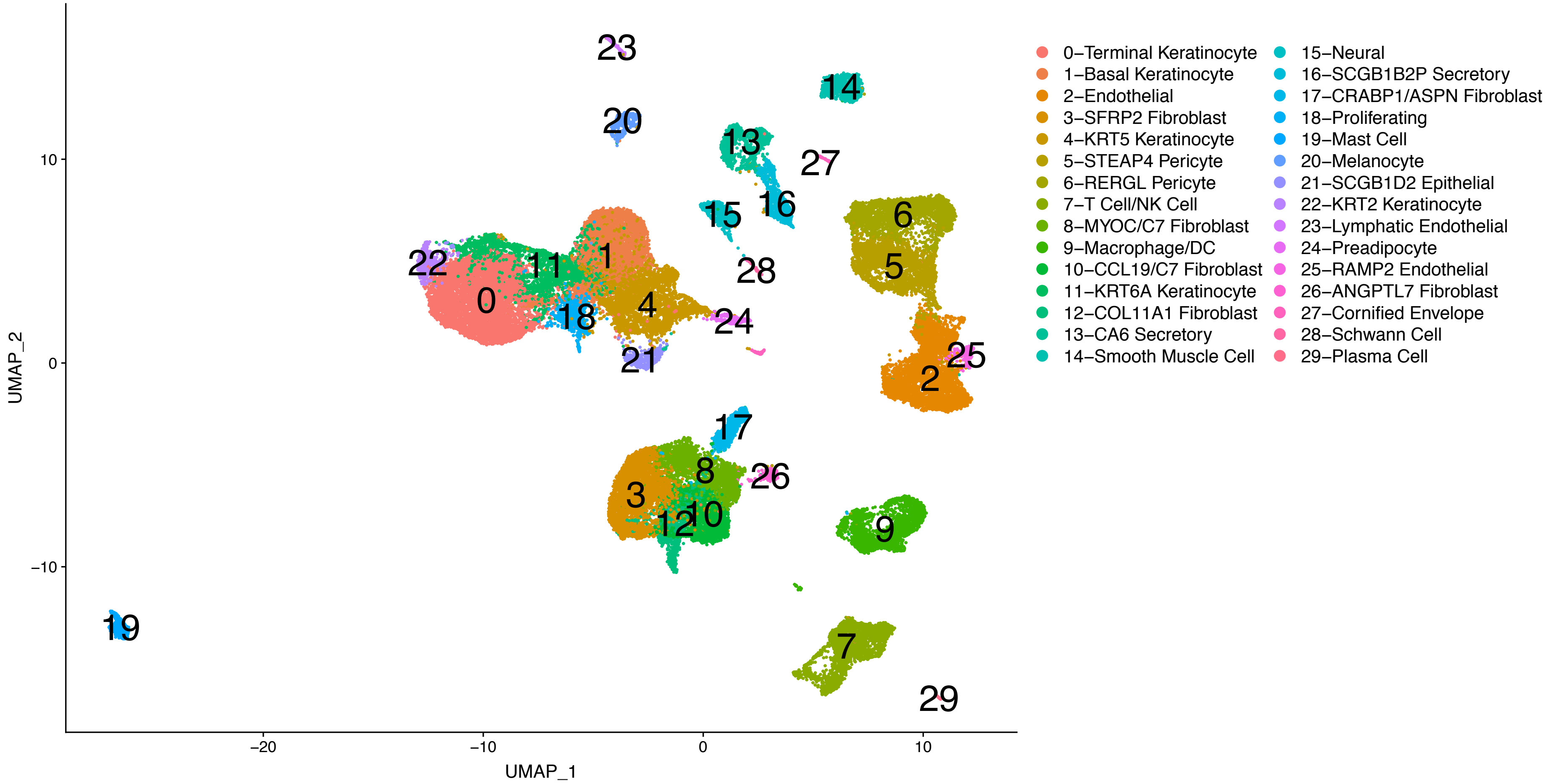

Supplementary Figure 9. UMAP plot of scRNA-seq data from control and SSc skin biopsies. Transcriptomes of all cells obtained after enzymatic digestion of dorsal mid-forearm skin biopsies from 10 healthy control and 12 SSc subjects, showing each SLM cluster by color.

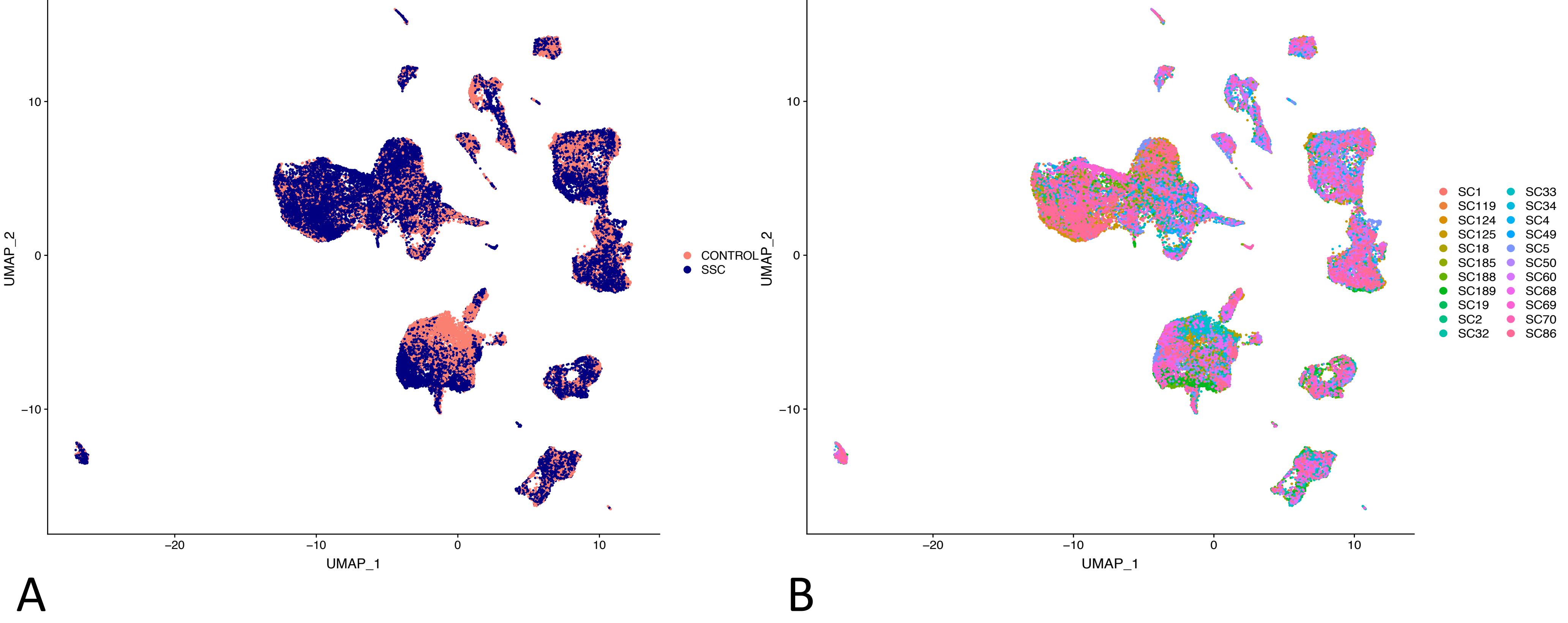

Supplementary Figure 10. UMAP plot of scRNA-seq data from control and SSc skin biopsies. Transcriptomes of all cells obtained after enzymatic digestion of dorsal mid-forearm skin biopsies from 10 healthy control and 12 SSc subjects, showing control and SSc by color (panel A; P=pink=control, blue=SSc or by patient samples (panel B, sample ID indicated to right)..

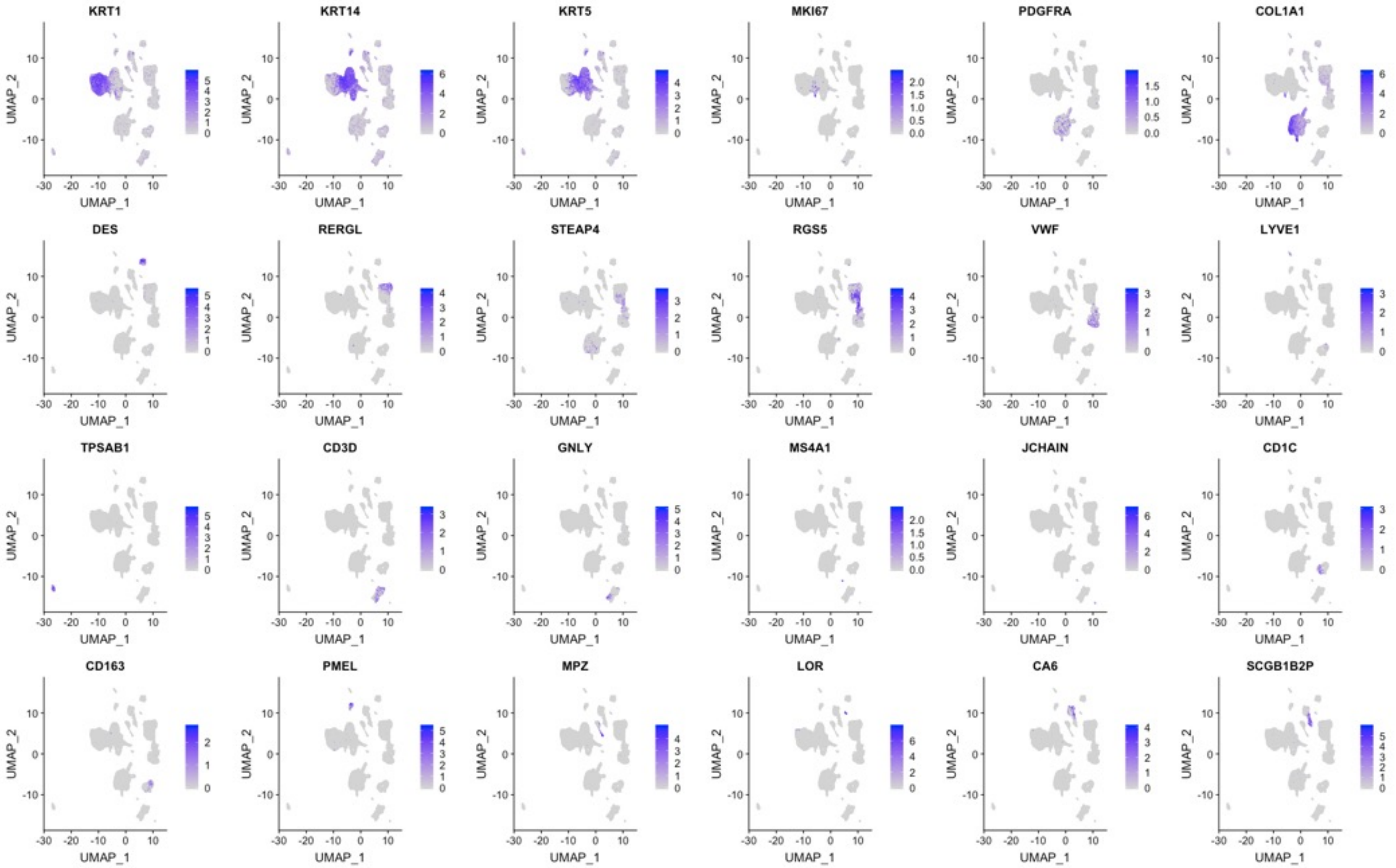

Supplementary Figure 11. UMAP feature plots showing marker genes for each cell type in combined normal and SSc skin analysis. T-SNE plots are as in Figure 1. Purple coloration indicates the level of expression.

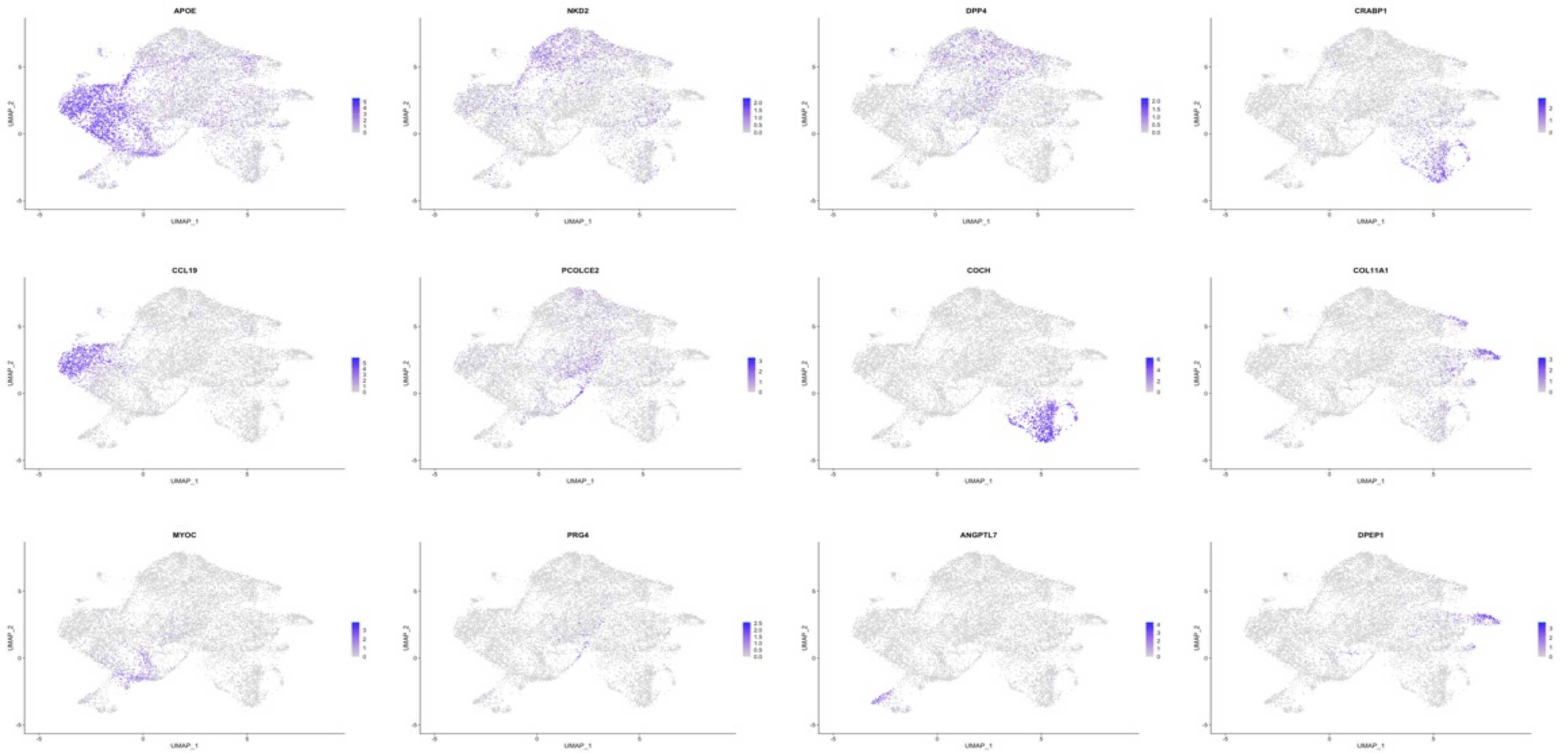

Supplementary Figure 12. Feature plots of key marker genes and proportions of fibroblast subclusters from control healthy and SSc skin. Cells from combined analysis of healthy control (n=10 and SSc (n=12) mid-forearm skin biopsies. Purple indicates increased expression (panel A).

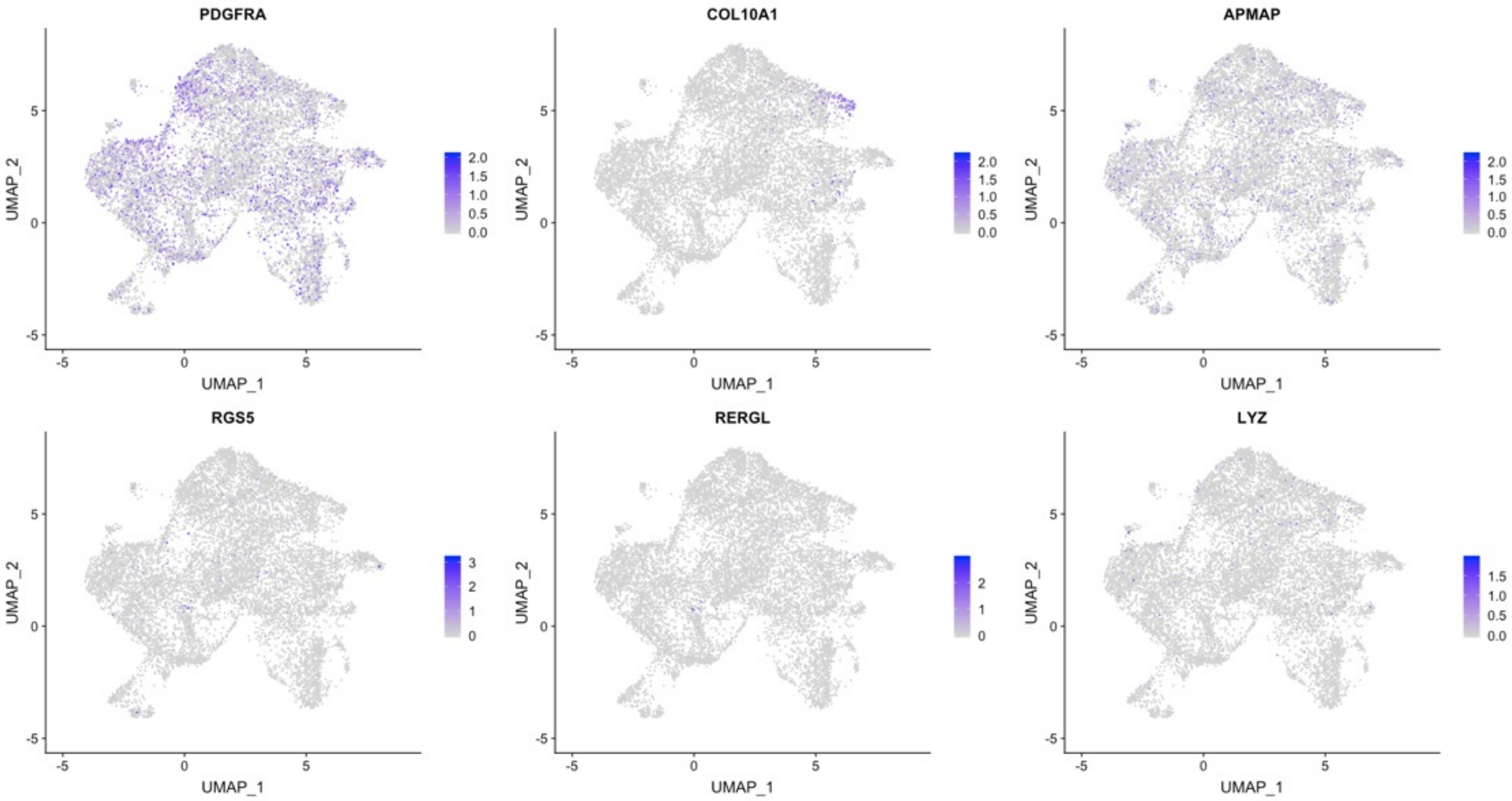

Supplementary Figure 13. Fibroblast cell clusters lack markers, suggesting preadipocyte, pericyte or myeloid progenitors. Fibroblast cell clusters (clusters 3, 8, 10, 12, 17) lack adipocyte (AMPAP, cluster 24); pericyte (RERGL and RGS5, clusters 2, 5 and 6) and myeloid cell markers (LYZ, cluster 9; feature plots, panel A; violin plots, panel B). Fibroblast subclusters also show a lack of any subset of fibroblasts expressing peradipocyte, pericyte or myeloid markers (panel C).

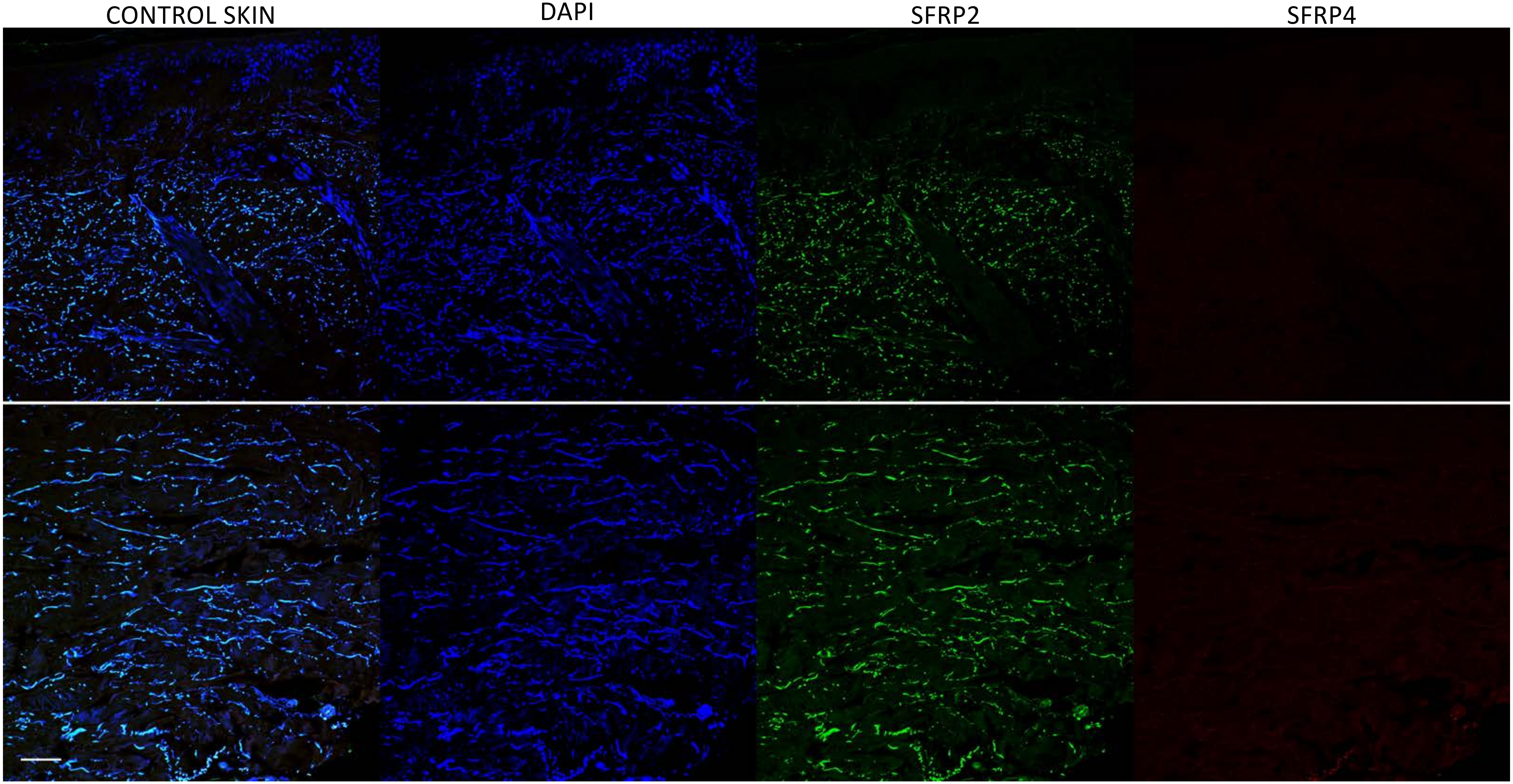

Supplementary Figure 14A . Immunofluorescent staining of control skin. Dual IF staining is shown of SFRP2 (green) and SFRP4 (red). SFRP2 expressing fibroblasts are visible in the papillary dermis (upper panels) and more so in the reticular dermis (lower panels). SFRP4 (red) expression was not detected in control skin. Staining representative of n=5. Scale bar=100 μM.

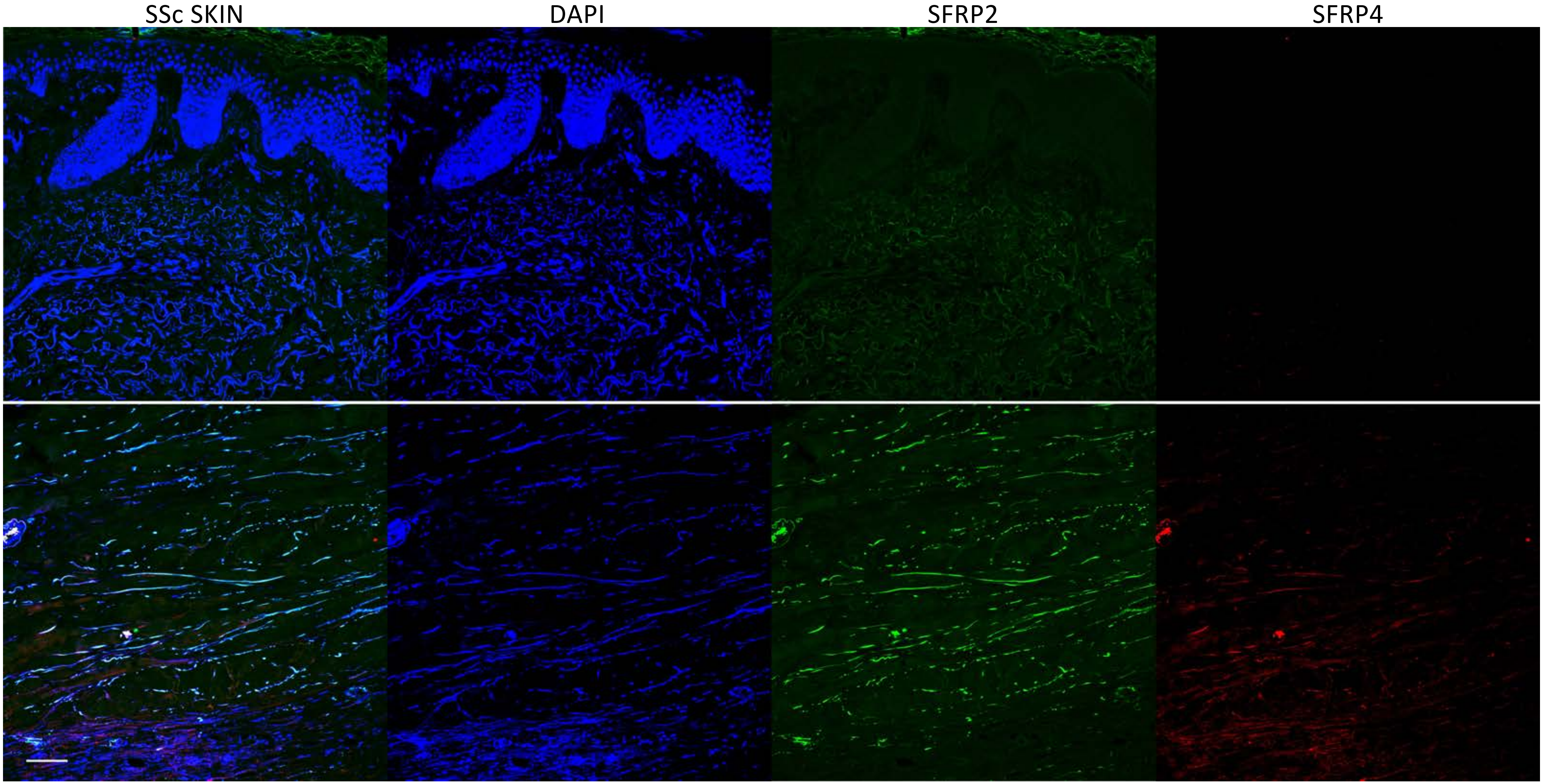

Supplementary Figure 14B. Immunofluorescent staining of SSc skin. Dual IF staining is shown of SFRP2 (green) and SFRP4 (red). SFPR2 expressing fibroblasts are visible in the reticular dermis (lower panels). A smaller population of dual expressing SFRP2 (green) and SFRP4 (red) cells were detected the reticular dermis (lower panels) of SSc skin. Staining representative of n=5 . Scale bar=100 μM.

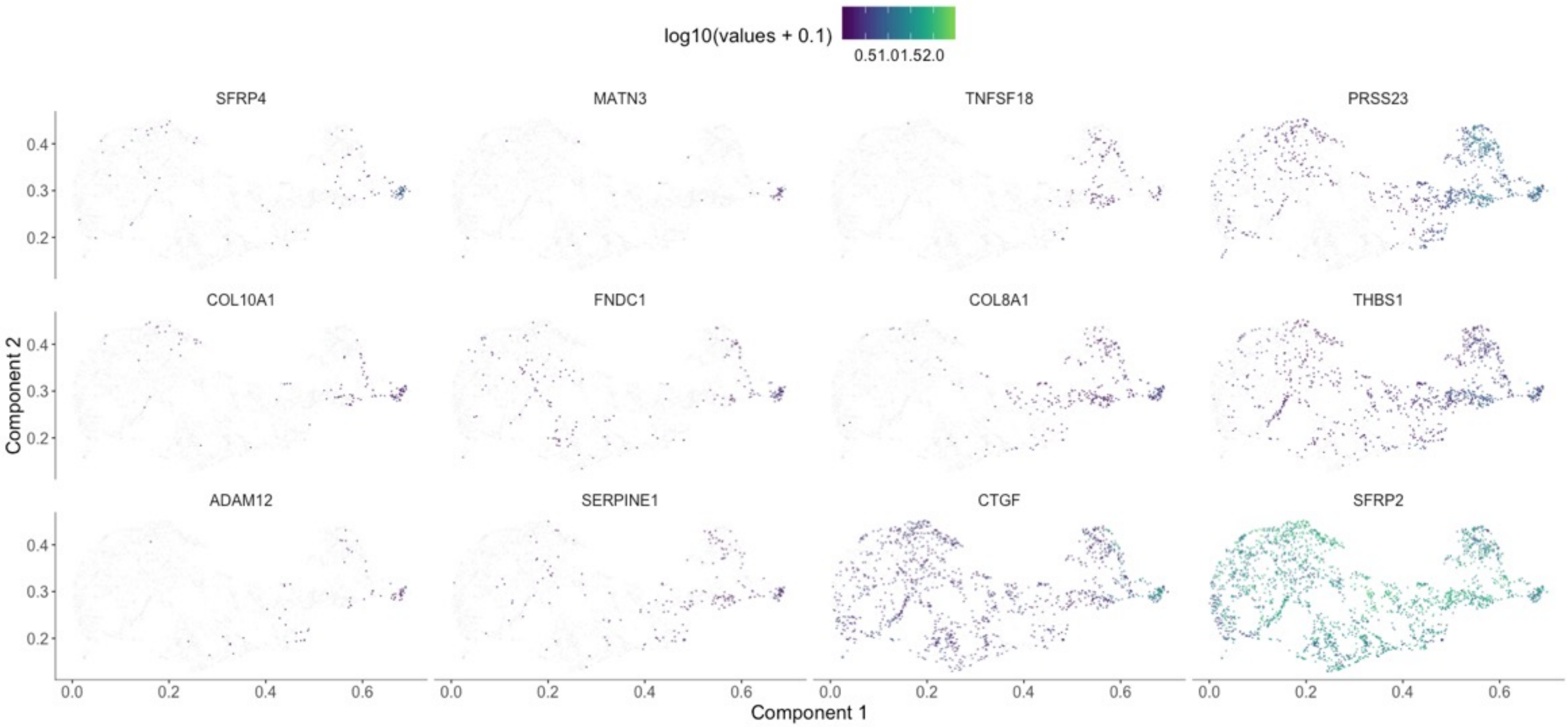

C

Supplementary Figure 15. Pseudotime modeling of SFRP2+ fibroblast differentiation in SSc skin. Fibroblast subclusters defined in Figure 2 were analyzed using Monocle with the trajectory as indicated by the black line, with cells colored by subcluster of origin: subclsuter 1 (red), subcluster 3 (green) and subcluster 4 (blue; panel A) or by subject status: healthy control (red) and SSc (blue; panel B). SFRP2 was expressed by all of the cells (panel C, right lower panel). PRSS23, THBS1, TNFSF18, and COL8A1 were expressed more highly by cells clustered later in pseudotime, corresponding to fibroblast subcluster 4 (panel C). SFRP4, COL10A1, ADAM12, MATN3, FNDC1, SERPINE1 and CTGF were more highly expressed even later in pseudotime, corresponding to myofibroblasts identified in t-SNE plots (Figures 4 and 5).

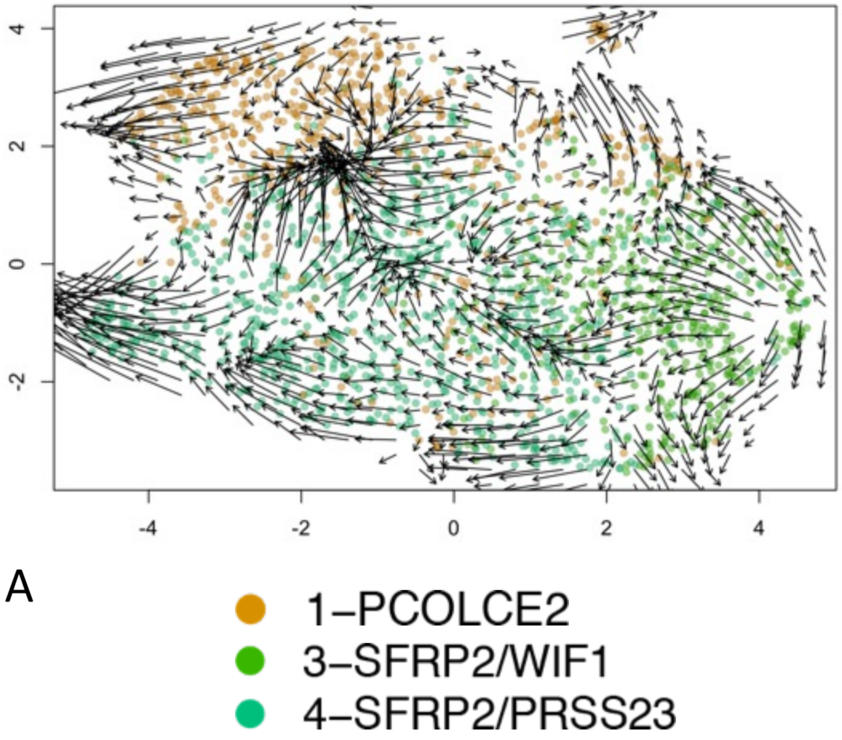

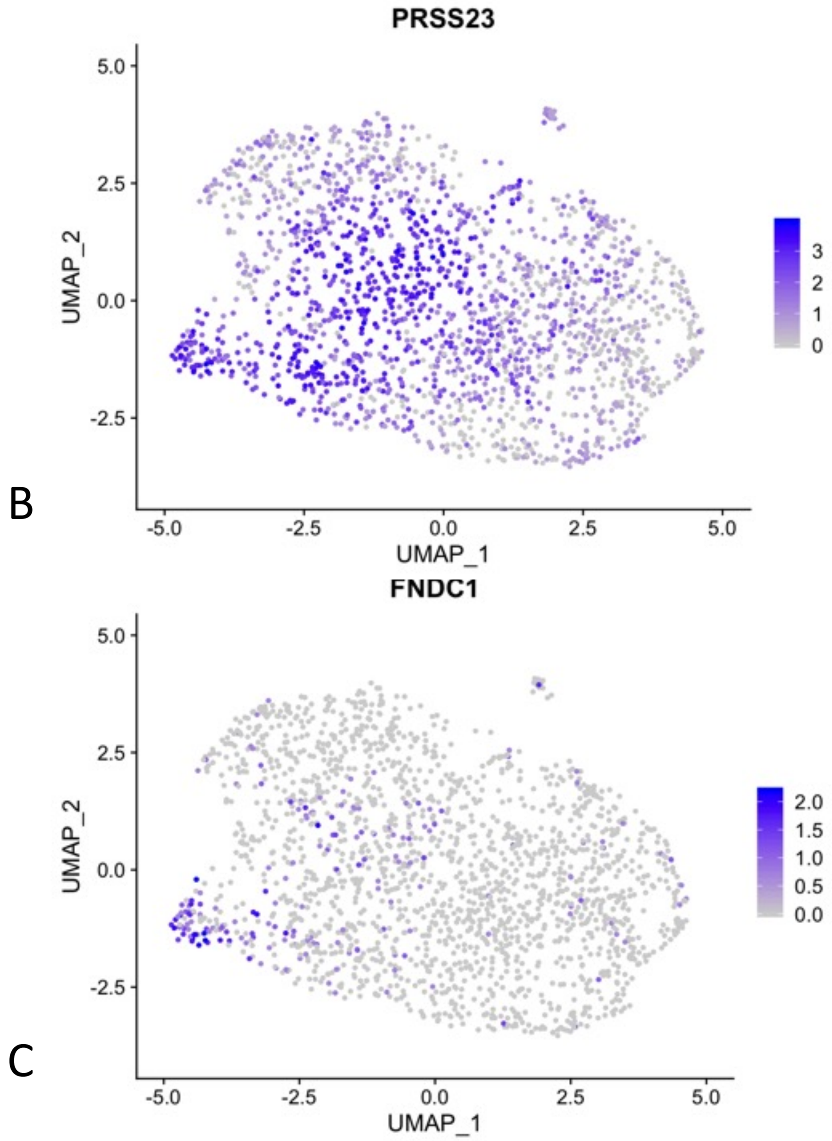

Supplementary Figure 16. UMAP projection of Velocyto analysis.
UMAP projection of Velocyto analysis of SFRP2+ clusters 1, 3 and 4, arrows indicating the direction of unspliced to spliced mRNAs (panel A). Markers of SSc fibroblasts (PRSS23, panel B) and myofibroblasts (FNDC1, panel C) are highlighted in purple.

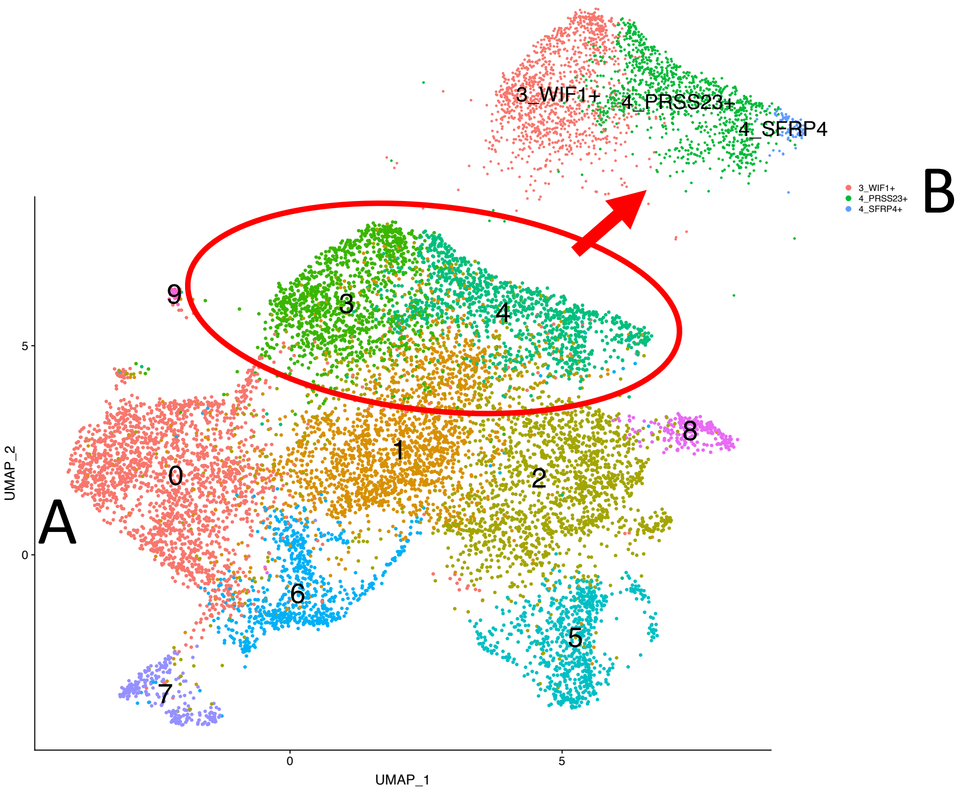

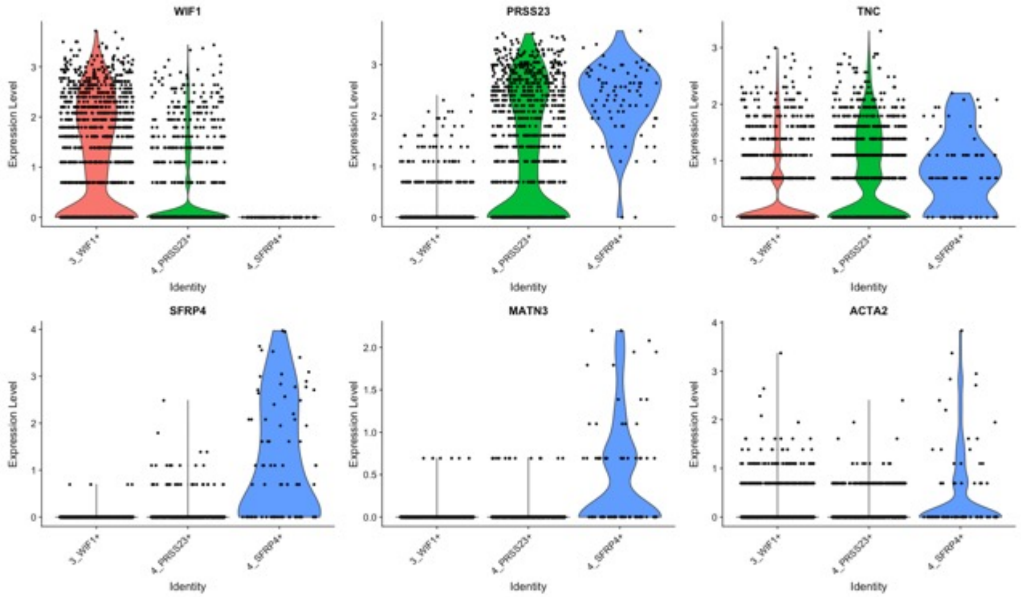

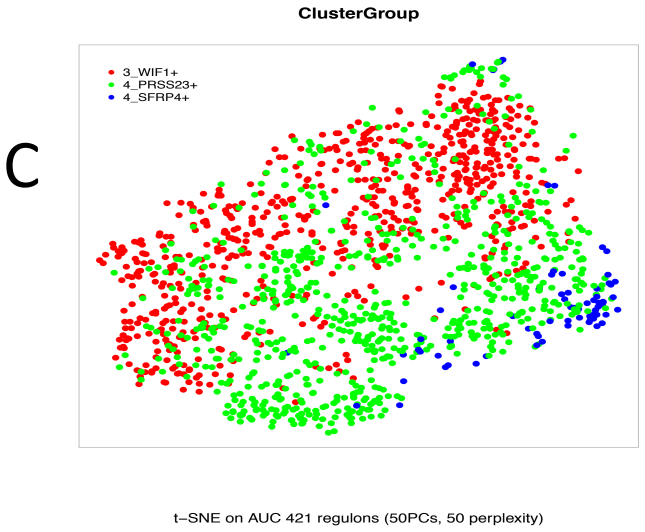

Supplementary Figure 17. Analysis of myofibroblast regulons. SFRP4-expressing cells (myofibroblasts) in cluster 4 were separated as indicated (panel A, showing expression of marker genes for each subcluster (panel B) and then analyzed by T-SNE by regulon (panel C)

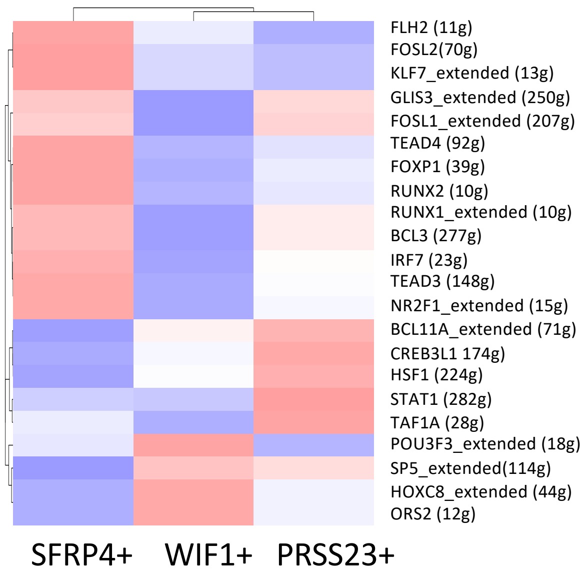

Supplementary Figure 18. Clustering of SFRP2+ regulons. Clustering of regulons comparing SFRP2+WIF1+ cluster 3 (WIF1+) with subcluster 4 divided into myofibroblasts (SFRP4+) and SFRP2+PRSS23+WIF1- non-myofibroblasts (PRSS23+).

Supplementary Figure 19. Analysis of regulons using filtered gene expression data. SFRP4-expressing cells (myofibroblasts) in cluster 4 were separated as in Figure S13, panel A, analyzed by T-SNE by regulon (panel A). Compressed clustering of TFs associated with each regulon (panel B), analyzing genes after filtering out genes expressing less than 6 UMI in all cells or expressed in less than 1% of the cells from SFRP2+ control cells (mainly SFRP2hiWIF1+, subcluster 3 cells; WIF1+), SFRP2+ SSc cells, (mainly SFRP2hiPRSS23+WIF1-, subcluster 4 cells; PRSS23+), and myofibroblasts (SFRP2hiSFRP4+ cells; SFRP4+). Amplified region (panels C) as indicated on the compressed panel

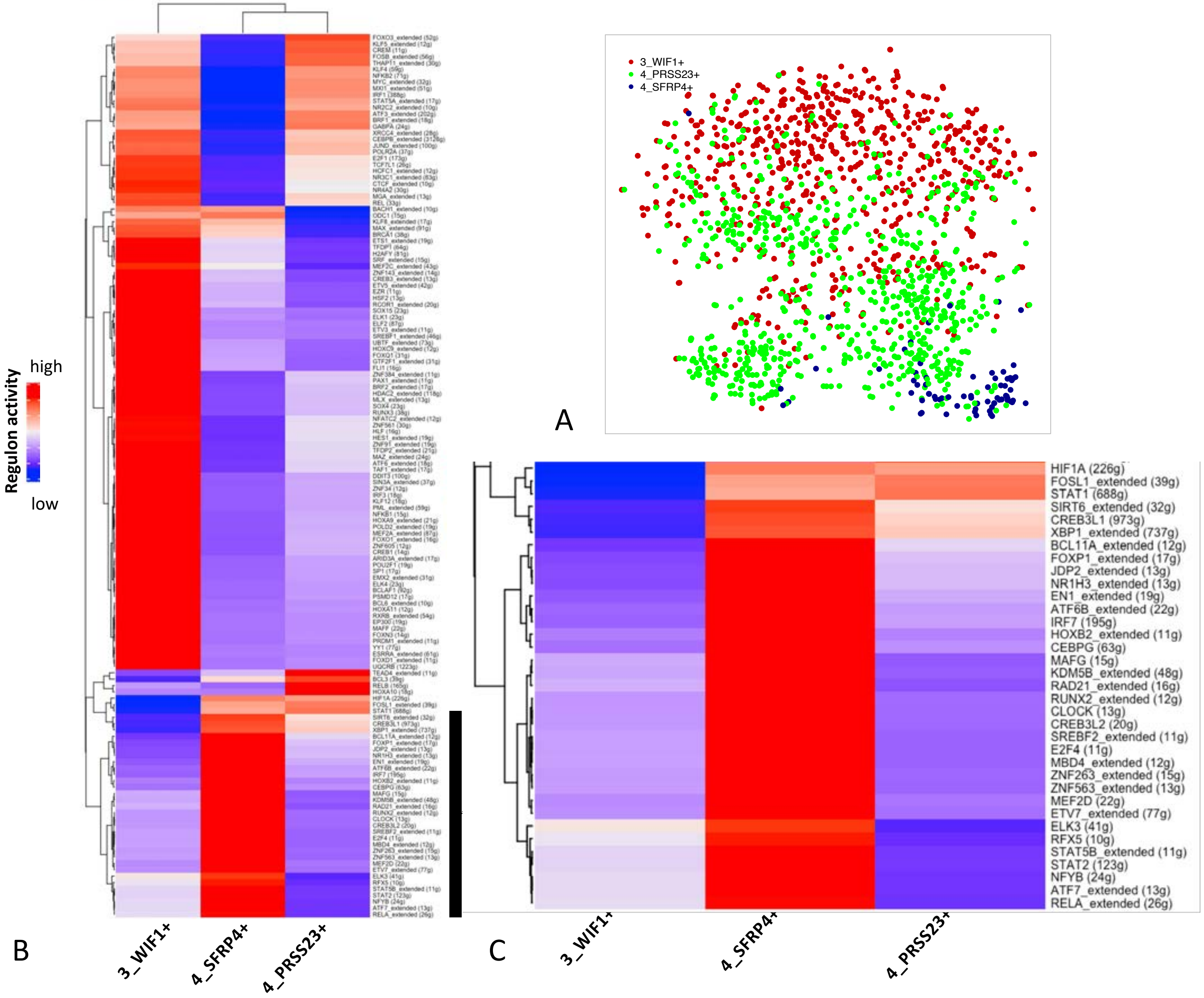

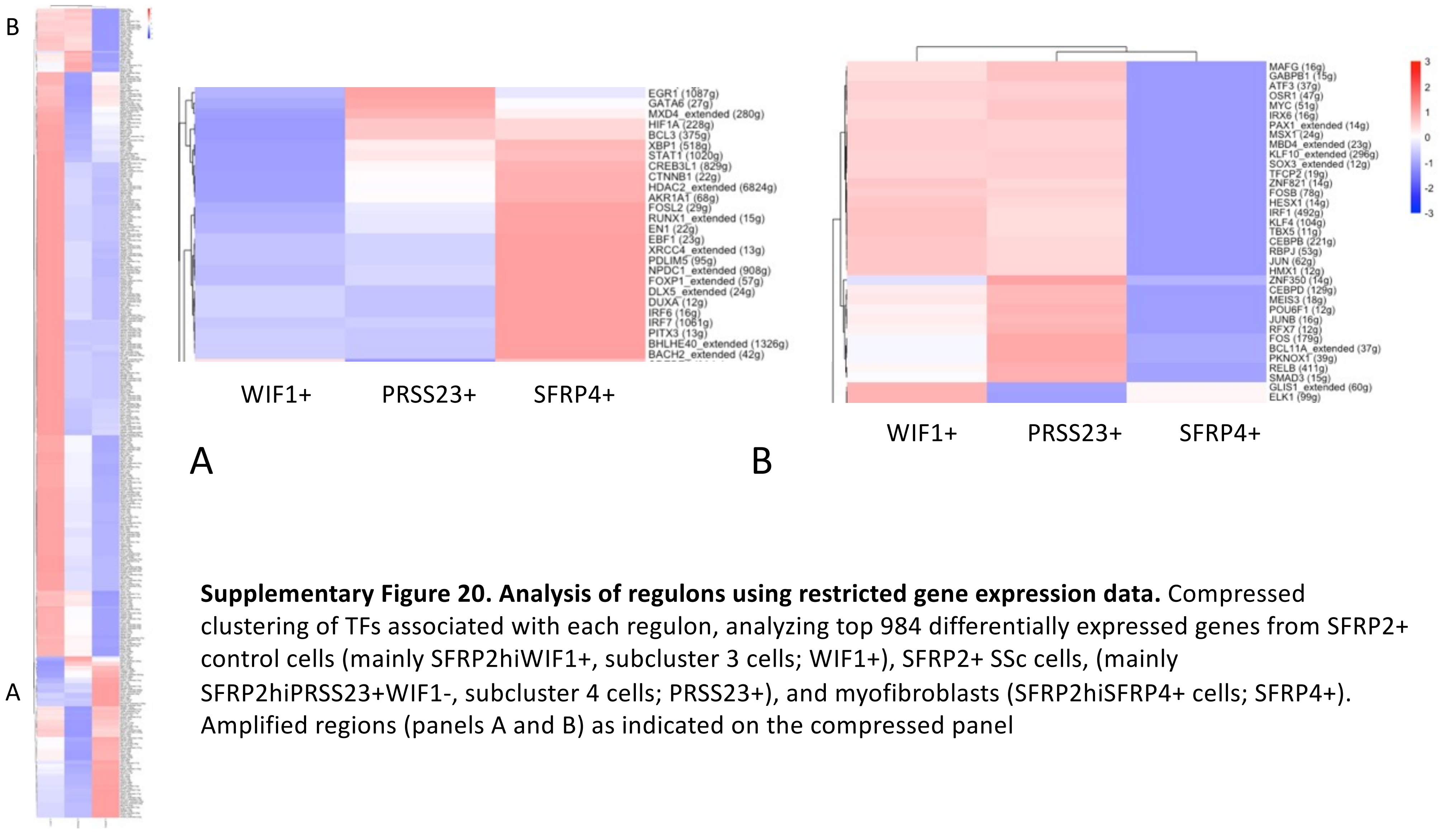

Supplementary Figure 20. Analysis of regulons using restricted gene expression data. Compressed clustering of TFs associated with each regulon, analyzing top 984 differentially expressed genes from SFRP2+ control cells (mainly SFRP2hiWIF1+, subcluster 3 cells; WIF1+), SFRP2+ SSc cells, (mainly SFRP2hiPRSS23+WIF1-, subcluster 4 cells; PRSS23+), and myofibroblasts (SFRP2hiSFRP4+ cells; SFRP4+). Amplified regions (panels A and B) as indicated on the compressed panel

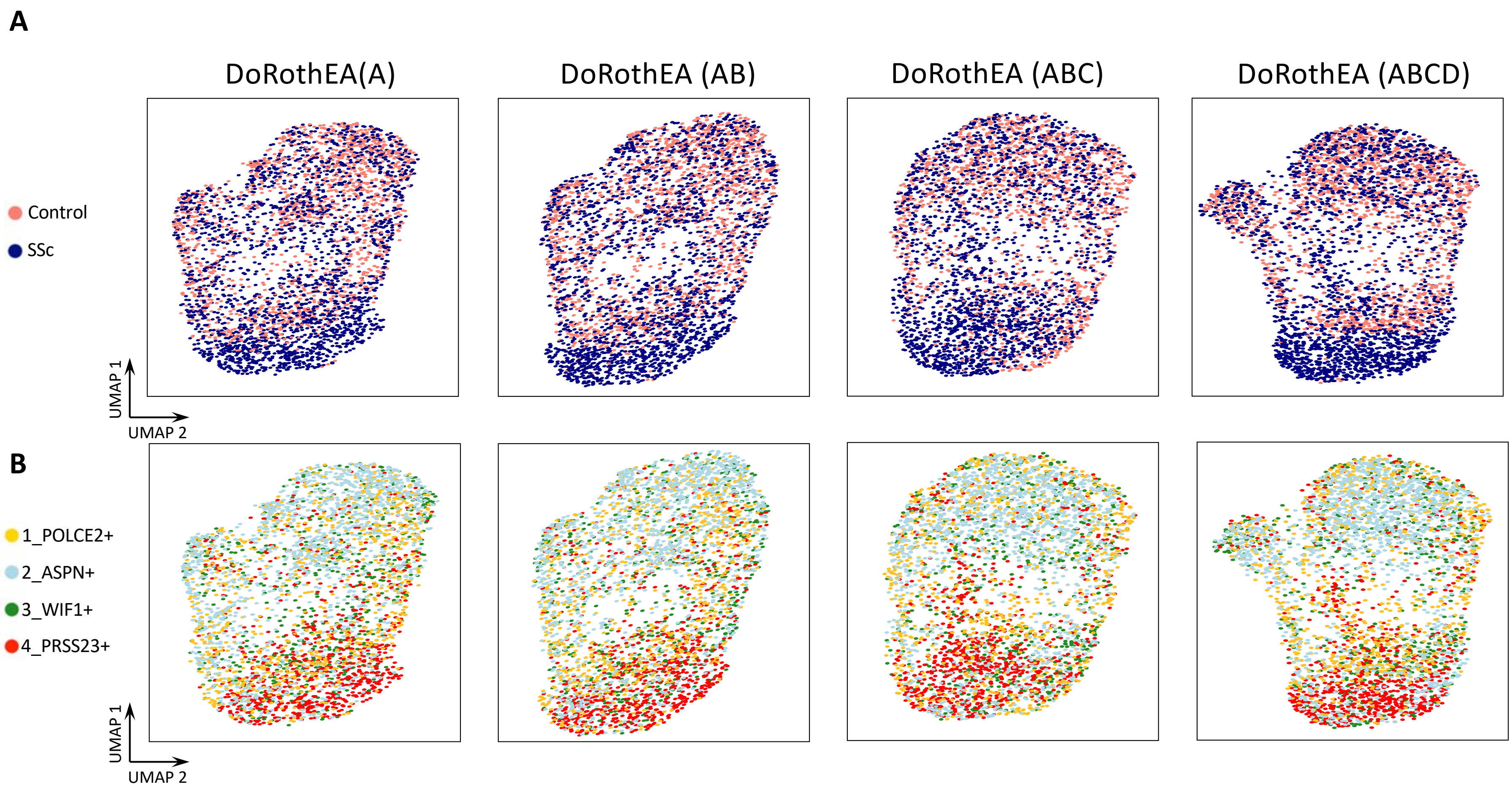

Supplementary Figure 21. (see legend following).

C

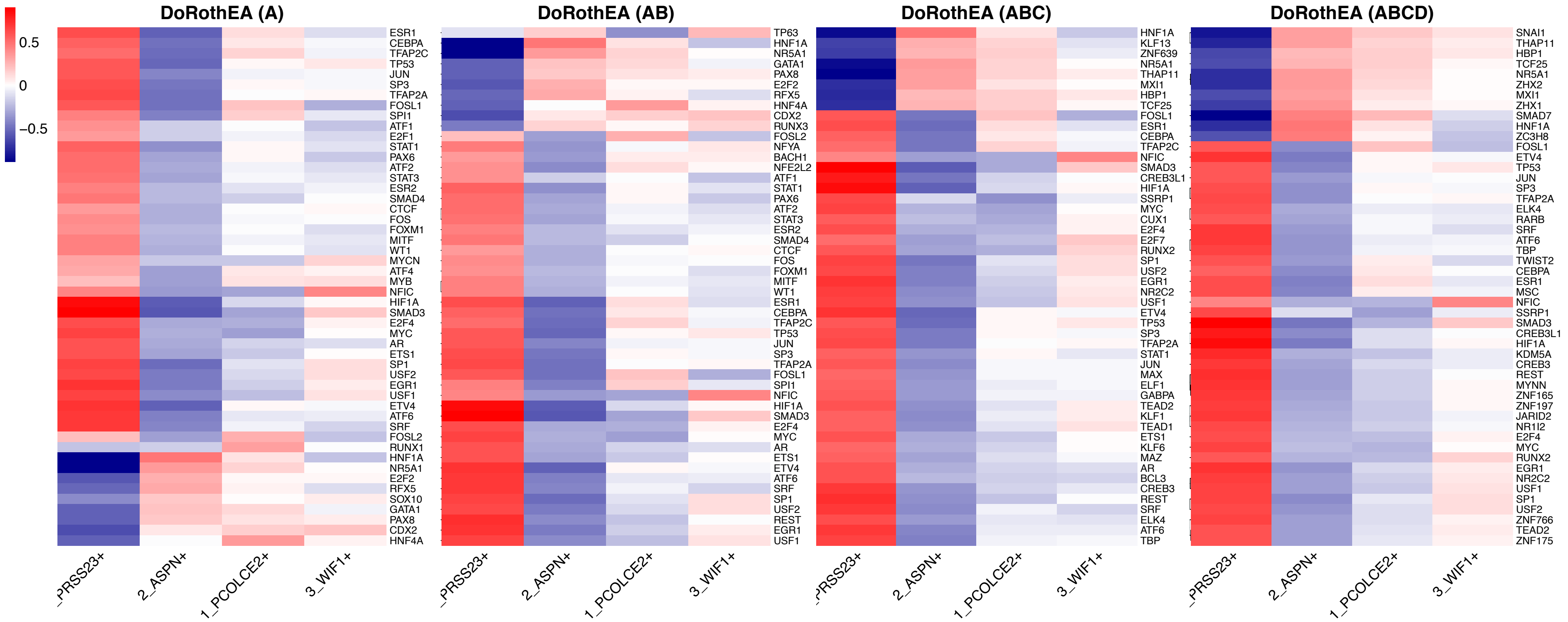

Supplementary Figure 21. Foot-printed based regulon analysis by DoRothEA with high to low confidence levels of TF-target interactions. (A, B) UMAP plots of regulons clustering colored by gene expression clusters in (A), and by SSc/healthy disease status in (B). (C) Compressed clustering of TF-target interactions in cluster 1_PCOLCE2+, 2_ASPN+, 3_WIF1+ and 4_PRSS23+. DoRothEA comprises five confidence levels of TF-target iterations: Level A are interactions that are supported by all four lines of evidence, manually curated by experts in specific reviews, or supported both in at least two curated resources; Level B-D are reserved for curated and/or ChIP-seq interactions with different levels of additional evidence.

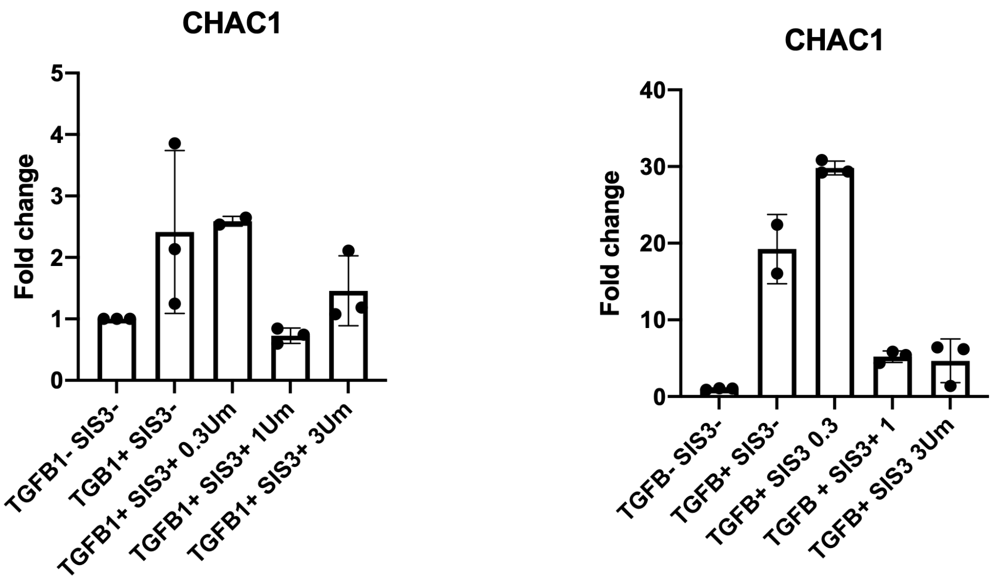

Supplementary Figure 22. CHAC1 regulation through Smad3 phosphorylation. Dermal fibroblast cultures from one control (left panel) and one SSc (right panel) subject were pretreated for one hour with SIS3 at concentrations indicated with or without TGFβ-1 2 ng/ml for 14-16 hours. CHAC1 mRNA expression was determined by qRT-PCR (n=3 replicates).

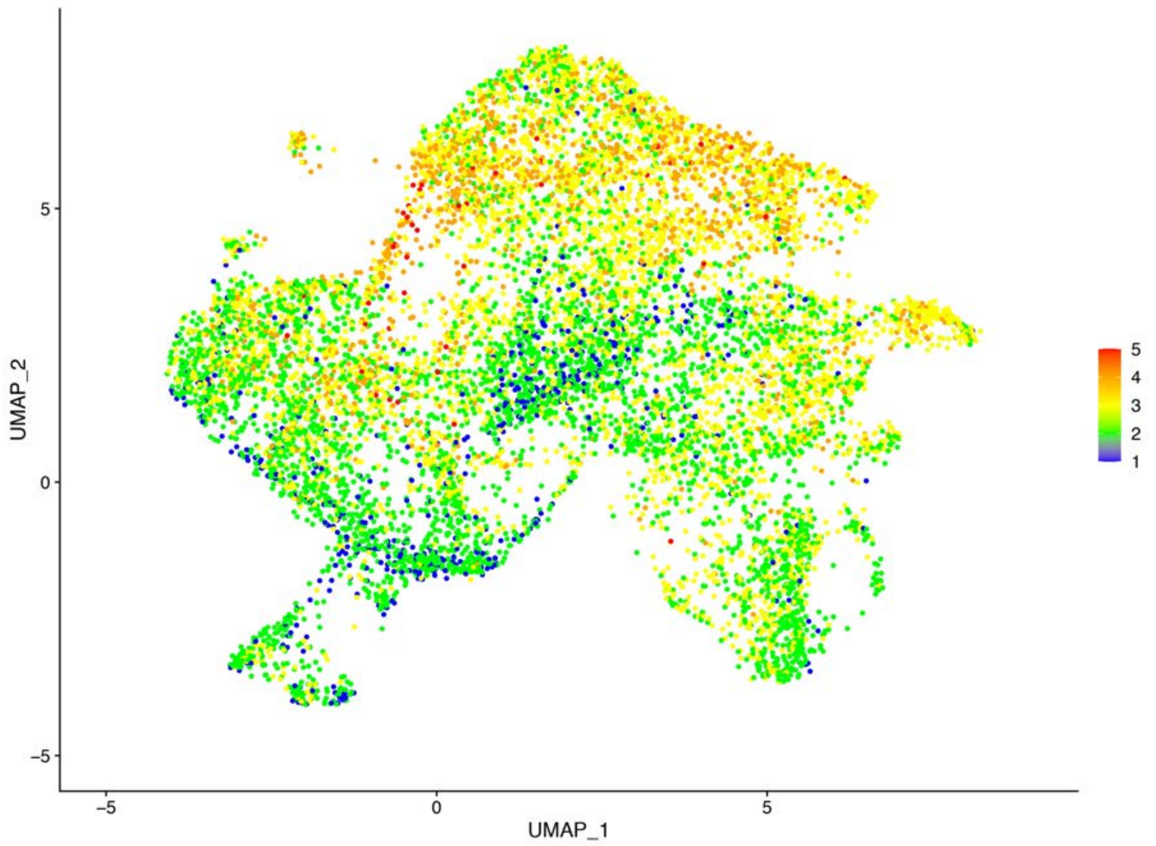

Supplementary Figure 23. Feature plot showing module expression derived from DoRothEA. Feature plot showing module expression derived from DoRothEA SMAD3 regulated genes (33 genes, left panel).

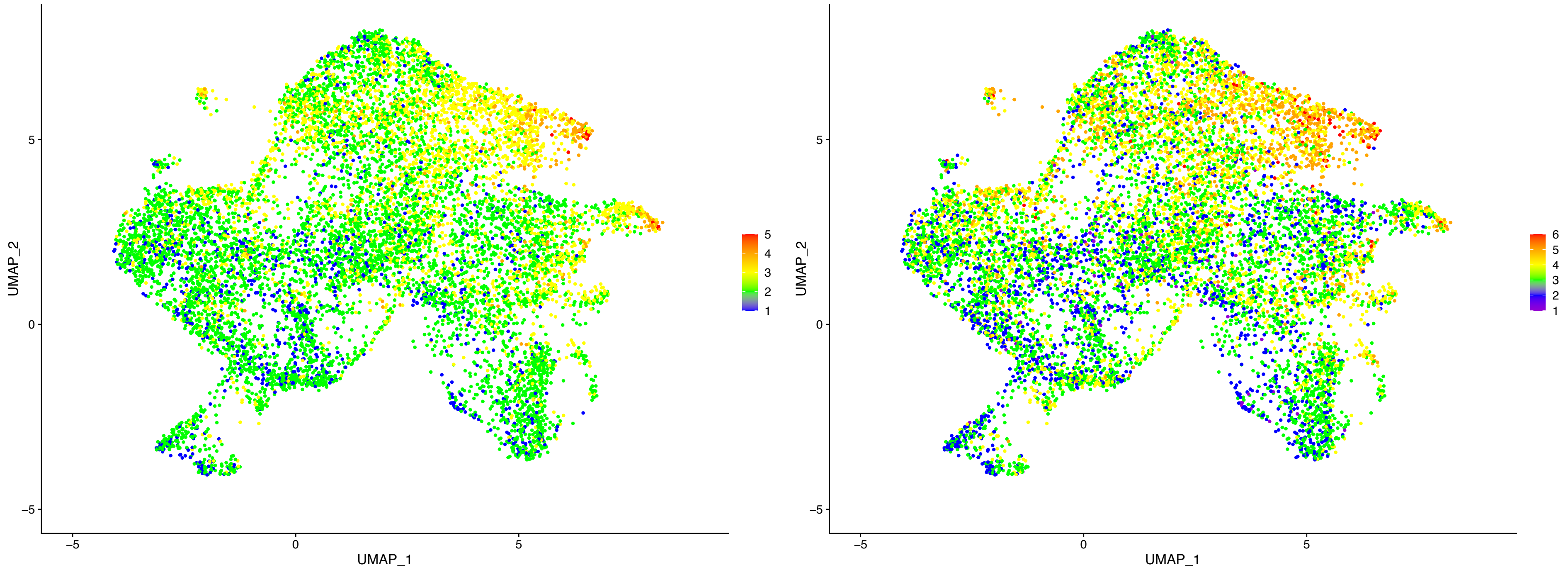

Supplementary Figure 24. Feature plots showing module expression derived from SMAD3 siRNA treated myofibroblasts. Feature plots showing module expression derived from SMAD3 siRNA treated myofibroblasts, filtered for gene expression, genes <50 TPM filtered out showing expression less than 0.8 of untreated control, excluding genes showing expression less than 0.7 of HRPT1 compared to non-targeting control (415 genes, left panel); or genes <100 TPM filtered out showing expression less than 0.7 of untreated control, excluding genes showing expression less than 0.7 of HRPT1 compared to non-targeting control (74 genes).
