# Supplementary material for: Myofibroblast transcriptome indicates SFRP2hi fibroblast progenitors in systemic sclerosis skin
Source: Nat Commun. 2021 Jul 19;12:4384. doi: 10.1038/s41467-021-24607-6 (PMC8289865; doi:10.1038/s41467-021-24607-6)
Supplement: Supplementary file 3 — Supplementary Data Legends [file 41467_2021_24607_MOESM3_ESM.docx]

File Name: Supplementary Data 1

Description: Most differentially expressed genes in normal fibroblast clusters.

File Name: Supplementary Data 2

Description: Subject Demographics

File Name: Supplementary Data 3

Description: Top 50 most highly differentially expressed genes in each skin cluster

File Name: Supplementary Data 4

Description: Average expression of genes in each cluster comparing healthy and SSc cells

File Name: Supplementary Data 5

Description: Top 50 most highly differentially expressed genes in each fibroblast subcluster

File Name: Supplementary Data 6

Description: GO terms associated with fibroblast subcluster 4

File Name: Supplementary Data 7

Description: Genes differentially expressed between cluster 4 compared to cluster 3 SFRP2hi fibroblasts

File Name: Supplementary Data 8

Description: Gene expression in control and SSc fibroblast subclusters

File Name: Supplementary Data 9

Description: Average log fold change (SFRP4+/SFRP4-)

File Name: Supplementary Data 10

Description: Gene regulation by TGF-b1, TGF-b2, TGF-b3

File Name: Supplementary Data 11

Description: RNA-seq results showing TPM and fold change comparing myofibroblasts treated with SMAD3 siRNA, or HPRT1 siRNA to non targeting siRNA

File Name: Supplementary Data 12

Description: Primers for Taq-man PCR
