# Supplementary material for: Myofibroblast transcriptome indicates SFRP2hi fibroblast progenitors in systemic sclerosis skin
Source: Nat Commun. 2021 Jul 19;12:4384. doi: 10.1038/s41467-021-24607-6 (PMC8289865; doi:10.1038/s41467-021-24607-6)
Supplement: Supplementary file 16 — Reporting Summary [file 41467_2021_24607_MOESM16_ESM.pdf]

nature research

Corresponding author(s): Robert Lafyatis, MD
Last updated by author(s): Jun 1, 2021

Reporting Summary

Nature Research wishes to improve the reproducibility of the work that we publish. This form provides structure for consistency and transparency in reporting. For further information on Nature Research policies, see our Editorial Policies and the Editorial Policy Checklist.

Statistics

For all statistical analyses, confirm that the following items are present in the figure legend, table legend, main text, or Methods section.

n/a	Confirmed	
☐	☒	The exact sample size (*n*) for each experimental group/condition, given as a discrete number and unit of measurement
☐	☒	A statement on whether measurements were taken from distinct samples or whether the same sample was measured repeatedly
☐	☒	The statistical test(s) used AND whether they are one- or two-sided<br>*Only common tests should be described solely by name; describe more complex techniques in the Methods section.*
☒	☐	A description of all covariates tested
☐	☒	A description of any assumptions or corrections, such as tests of normality and adjustment for multiple comparisons
☐	☒	A full description of the statistical parameters including central tendency (e.g. means) or other basic estimates (e.g. regression coefficient) AND variation (e.g. standard deviation) or associated estimates of uncertainty (e.g. confidence intervals)
☐	☒	For null hypothesis testing, the test statistic (e.g. *F*, *t*, *r*) with confidence intervals, effect sizes, degrees of freedom and *P* value noted<br>*Give P values as exact values whenever suitable.*
☒	☐	For Bayesian analysis, information on the choice of priors and Markov chain Monte Carlo settings
☐	☒	For hierarchical and complex designs, identification of the appropriate level for tests and full reporting of outcomes
☒	☐	Estimates of effect sizes (e.g. Cohen's *d*, Pearson's *r*), indicating how they were calculated

Our web collection on statistics for biologists contains articles on many of the points above.

Software and code

Policy information about availability of computer code

Data collection	CellRanger 3.0.2 for the alignment to the reference genome.<br>Seurat 3.0
Data analysis	Single-cell RNA-sequencing FASTQ files were generated and aligned to human reference genome GRCh38 using Cell Ranger (v3.0.2, 10X-Genomics) and subsequently analyzed using Seurat (v3.0.2) and SCTransform 0.1.0. Trajectory analysis was performed using Monocle 3 alpha. SCENIC (v1.1.1) was implemented to identify gene regulatory networks, transcription factor binding motifs, and cell states from single-cell RNA-seq data. The publicly available R software environment (v3.6) was used to perform these analyses.

For manuscripts utilizing custom algorithms or software that are central to the research but not yet described in published literature, software must be made available to editors and reviewers. We strongly encourage code deposition in a community repository (e.g. GitHub). See the Nature Research guidelines for submitting code & software for further information.

Data

Policy information about availability of data

All manuscripts must include a data availability statement. This statement should provide the following information, where applicable:
- Accession codes, unique identifiers, or web links for publicly available datasets
- A list of figures that have associated raw data
- A description of any restrictions on data availability

The accession number for the single cell RNAseq data is: GSE138669 this dataset was made public on 5/20/21

https://www.ncbi.nlm.nih.gov/geo/query/acc.cgi?acc=GSE138669

Field-specific reporting

Please select the one below that is the best fit for your research. If you are not sure, read the appropriate sections before making your selection.

☒ Life sciences ☐ Behavioural & social sciences ☐ Ecological, evolutionary & environmental sciences

For a reference copy of the document with all sections, see nature.com/documents/nr-reporting-summary-flat.pdf

Life sciences study design

All studies must disclose on these points even when the disclosure is negative.

Sample size	Sample size was deemed sufficient based on the detection of disease specific cell populations.
Data exclusions	Data were filtered for level of mRNA expression, Raw data were filtered for minimum count of 200 genes expressed per cell. Raw data was also filtered for cell Mitochondrial gene %. This techinques is used to remove dead and dying cells form the dataset. This is a previously established data filtering technique by Satija lab which created the Seurat software package.
Replication	We have included analysis of a second dataset described in the manuscript. Reproducibility of single cell data was assured by study of 10 normal and 12 SSc skin samples, the reproducibility between samples indicated in Figure 1b ad 2b. Reproducibility of immunohistochemistry has been added to each figure legend as requested.
Randomization	Allocation was based on patient willingness to participate and availability.
Blinding	This was not an interventional study. The data analysis was performed in a blinded fashion.

Reporting for specific materials, systems and methods

We require information from authors about some types of materials, experimental systems and methods used in many studies. Here, indicate whether each material, system or method listed is relevant to your study. If you are not sure if a list item applies to your research, read the appropriate section before selecting a response.

Materials & experimental systems

n/a	Involved in the study	
☐	☒	Antibodies
☒	☐	Eukaryotic cell lines
☒	☐	Palaeontology and archaeology
☒	☐	Animals and other organisms
☐	☒	Human research participants
☐	☒	Clinical data
☒	☐	Dual use research of concern

Methods

n/a	Involved in the study	
☒	☐	ChIP-seq
☒	☐	Flow cytometry
☒	☐	MRI-based neuroimaging

Antibodies

Antibodies used

Monoclonal mouse anti-SMA (1:1000; Dako, Denmark AS, Denmark; M0851; Clone14A)
Polyclonal rabbit anti-CCL19 (1:500; Abcam, USA; ab221704; GR3331458-1)
Monoclonal mouse anti-CRABP1 (1:500; ThermoFisher, USA; MA3-813; C-1)
Polyclonal rabbit anti-POSTN (1:250; Abcam, USA; ab14041; GR3178086-8)
Monoclonal mouse anti-SLPI (1:50; Abcam, USA; ab17157; 31; GR3304812-1)
Monoclonal mouse SFRP2 (1:250; Millipore, USA; MAB539; 80.8.6; 2800493)
Polyclonal rabbit SFRP4 (1:500; Proteintech, USA; 15328-1-AP)

Validation

These antibodies are all commercially available, validated by the manufacturer for reactivity in human tissue with immunohistochemical staining.
Monoclonal mouse anti-SMA (1:1000; Dako, Denmark AS, Denmark; M0851; Clone14A) IHC validation and specificity testing performed by Dako on Tissue Human Appendix or Human Tonsil with Fixation FFPE-Formalin Fixed Paraffin Embedded IHC, Titer 1:400-1:800
Polyclonal rabbit anti-CCL19 (1:500; Abcam, USA; ab221704; GR3331458-1) abcam validates the use of ab221704 in the following tested applications. The application notes include recommended starting dilutions; optimal dilutions/concentrations should be determined by the end user. IHC-P 1/500 - 1/1000. Perform heat mediated antigen retrieval with citrate buffer pH 6 before commencing with IHC staining protocol.
Monoclonal mouse anti-CRABP1 (1:500; ThermoFisher, USA; MA3-813; C-1) This Antibody was verified by Cell treatment to ensure that the antibody binds to the antigen stated.
Polyclonal rabbit anti-POSTN (1:250; Abcam, USA; ab14041; GR3178086-8) abcam validates the use of ab14041 in the following

tested applications. Recommended starting dilutions IHC-P 1/100 - 1/1000.; ICC/IF optimal dilutions/concentrations should be determined by the end user.

Monoclonal mouse anti-SLPI (1:50; Abcam, USA; ab17157; 31; GR3304812-1) abcam validates the use of ab17157 in the following tested applications IHC-Fr 1/50.
Monoclonal mouse SFRP2 (1:250; Millipore, USA; MABC539; 80.8.6; 2800493) Millipore validated this antibody by immunohistochemistry in human prostate and human breast cancer tissue. Immunohistochemistry Analysis: A 1:500 dilution of this antibody detected SFRP2 in human prostate and human breast cancer tissue.

Polyclonal rabbit SFRP4 (1:500; Proteintech, USA; 15328-1-AP) was validated by Proteintech by positive WB detected in HEK-293 cells, HeLa cells and Positive IHC detected in human breast cancer tissue, human skin cancer tissue at IHC 1:5—1:500. Suggested antigen retrieval with TE buffer pH 9.0; (*) Alternatively, antigen retrieval may be performed with citrate buffer pH 6.0

Further validation was performed with each antibody using control tissues and antibodies at a wide range of concentrations until optimal staining conditions were identified and reported in the methods section of this manuscript.

Human research participants

Policy information about studies involving human research participants

Population characteristics	Skin from systemic sclerosis patients was compared to healthy control skin samples The covariate relevant populations characteristics were balanced across sex, age, and total cell counts. This information is included in the results section of the manuscript.
Recruitment	Patients were recruited with early disease from a tertiary referral center.
Ethics oversight	University of Pittsburgh Institutional Review Board STUDY19040254

Note that full information on the approval of the study protocol must also be provided in the manuscript.

Clinical data

Policy information about clinical studies

All manuscripts should comply with the ICMJEguidelines for publication of clinical research and a completedCONSORT checklist must be included with all submissions.

Clinical trial registration	This is not a registered or interventional trial therefore not registered.
Study protocol	The I RB protocol: http://dom.pitt.edu/rheum/centers-institutes/scleroderma/ongoingclinicaltrials-scleroderma/
Data collection	The study recruitment and data collection began in June 1, 2016 and ended on February 12th 2018.
Outcomes	There was not a primary or secondary outcome measure as this was not a clinical trial. Single cell RNA-seq was assessed from subject skin biopsies
